# Efficacy of Chinese Herbal Formula Sini Zuojin Decoction in Treating Gastroesophageal Reflux Disease: Clinical Evidence and Potential Mechanisms

**DOI:** 10.3389/fphar.2020.00076

**Published:** 2020-02-27

**Authors:** Shaowei Li, Mengfen Huang, Guojing Wu, Weihan Huang, Zhanhui Huang, Xiaoqian Yang, Jinming Ou, Qipeng Wei, Chengli Liu, Shaoyuan Yu

**Affiliations:** ^1^ Guangzhou University of Chinese Medicine, Guangzhou, China; ^2^ Department of Otolaryngology, Guangdong Provincial Hospital of Traditional Chinese Medicine, Guangzhou, China; ^3^ Department of Endodontics, Guanghua School of Stomatology, Sun Yat-sen University, Guangzhou, China; ^4^ Department of Gastroenterology, Guangdong Provincial Hospital of Traditional Chinese Medicine, Guangzhou, China

**Keywords:** Sini Zuojin decoction, gastroesophageal reflux disease, Chinese herbal formula, systematic review, network pharmacology

## Abstract

**Background:**

Based on 122 cases reported in China, data mining indicated that Sini Powder (SNP) and the Zuojin Pill (ZJP) are both widely used as the basic recipe for treating Gastroesophageal Reflux Disease (GERD).

**Objectives:**

To evaluate the intervention effects of Sini Zuojin Decoction (SNZJD) in patients with GERD.

**Methods:**

A comprehensive collection of randomized controlled trials (RCTs) using SNZJD in patients with GERD that were published in domestic and foreign journals was made by computer retrieval. RevMan 5.3 software was used for meta-analysis and bias risk assessment, Stata 14.0 software was used for sensitivity analysis, GRADE profiler 3.6 was used to evaluate the level of evidence, and trial sequential analysis (TSA), employed to control for random errors, was performed to assess the main outcomes. Network pharmacology analysis was applied to preliminarily study the mechanisms of action of SNZJD on GERD.

**Results:**

Thirteen articles were eventually included, covering a total of 966 patients. Meta-analysis indicated that: ① the SNZJD plus traditional stomach medicines (SPTSM) group was more effective than the traditional stomach medicines (TSM) group (RR = 1.16, 95% CI [1.04, 1.29], P = 0.009); ② the experimental group with SNZJD was significantly better than TSM controls in improving heartburn, substernal chest pain, acid regurgitation, and food regurgitation symptoms (P < 0.0001); ③ SPTSM could significantly decrease total symptom scores with substantial effectiveness (P < 0.00001). The recurrence rate and adverse effects of SNZJD treatment were significantly reduced (P < 0.05). TSA showed that the effective rate of meta-analysis might be reliable, but the recurrence and safety results were still uncertain. According to the evaluation by the GRADE method, the quality of evidence was low. Besides, SNZJD might treat GERD by acting on related targets and pathways such as inflammation, hormone regulation, and so on.

**Conclusions:**

SNZJD might be useful in the treatment of GERD, but its long-term effects and specific clinical mechanisms are unclear. Due to the poor quality of the evidence, more samples and high-quality clinical studies should be tested and verified in the future.

## Introduction

Gastroesophageal reflux disease (GERD) (Zhang et al., 2017; Lin et al., 2019) refers to a comprehensive disease of reflux symptoms caused by reflux of gastric or duodenal content back up the esophagus or throat. It is usually diagnosed by the combination of clinical symptoms, reaction to acid suppression, objective endoscopy, and reflux monitoring (Cesario et al., 2018). Patients feel heartburn, acid regurgitation, post-sterna, nausea, and other discomforts; inconsistency between defense factors and infringement factors in the digestive system is the common pathogenic cause. Under endoscopic standards, GERD is mainly divided into three clinical types: non-erosive reflux disease (NERD), reflux esophagitis (RE), and Barrett esophagitis (BE). The pharmacological mechanism for treating GERD is the prevention of exposure to esophageal acid by inhibiting gastric acid secretion. The most important components of GERD management are interventions in lifestyle and treatments to reduce esophageal acid through local acid neutralization or inhibition of gastric acid secretions, or anti-reflux surgery (Hunt et al., 2017). At present, proton pump inhibitors (PPI) are used as first-line drugs, while gastrointestinal prokinetic agents (GPA) and mucosal protective agents (MPA) are also used to inhibit reflux. However, patients with long-term medication need to be alert and monitored for adverse drug reactions. Evidence (Hosseini et al., 2017) shows that even if PPI is gradually reduced during the course of treatment, the recurrence rate does not change much in the short term, whereas the corresponding gastrointestinal symptoms will increase, such as dyspepsia and irritable bowel syndrome. In addition, recent large-scale association studies based on the evaluation of prescription databases (Gyawali, 2017) have shown that the long-term use of PPI can lead to adverse reactions such as bacterial gastroenteritis, microscopic colitis, acute interstitial nephritis, fundic gland polyps, and hypomagnesemia. Therefore, some GERD patients suffering from gastrointestinal disorders have sought traditional Chinese medicine (TCM), especially medicinal herbs, for alternative treatment options (Teschke et al., 2015; Hosseinkhani et al., 2018).

**Figure 1:**
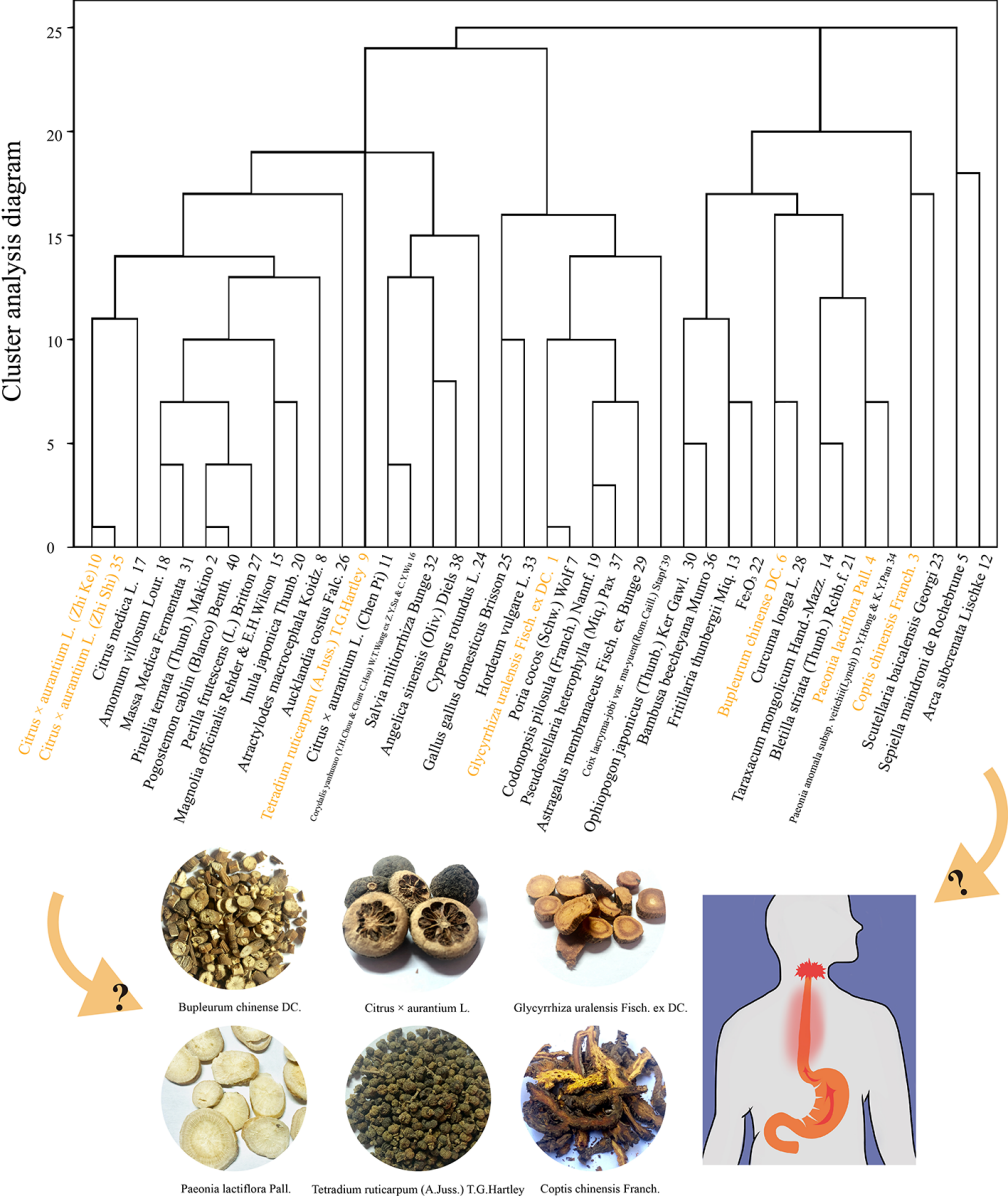
Cluster analysis of 122 cases of gastroesophageal reflux and the herb composition of SNZJD.

TCM is ancient oriental medicine. As a complementary and alternative medical treatment with fewer adverse effects and certain curative effects, the herbal formulae combined with fixed herbs are widely accepted and studied in China, Japan, South Korea, and other regions. In recent years, through the systematic review of reliable evidence, it has been found that classic TCM herbal formulae, such as Banxia Xiexin Decoction, Wendan Decoction, and others, can effectively improve GERD or some disorders of the gastrointestinal tract (Ling et al., 2015; Teschke et al., 2015; Tominaga and Arakawa, 2015; Dai et al., 2017; Xiao and Yang, 2018). Moreover, our team has collected 122 cases reported in the National Service Platform for Famous and Old Traditional Chinese Medicine (http://www.gjmlzy.com:83) and China National Knowledge Internet (http://www.cnki.net) *via* data mining based on using effect, flavor, meridian tropism, and syndrome differentiation to cluster analysis of tradition. Chinese herbal medicines with a analysis frequency of more than 15 times are shown in (**Table S1**). We have found that the famous veteran TCM doctors’ herbal formula usually included a combination decoction of Sini powder and Zuojin pill called “Sini Zuojin Decoction” (SNZJD), which, together, serves as the basic recipe for treating GERD. Therefore, in this study, we use SNZJD as a research prescription. The composition of the prescription is *Bupleurum chinense DC.* (Chai Hu, *Bupleuri Radix*), *Citrus × aurantium L.* (Zhi Shi, *Aurantii Fructus Immaturus*), *Paeonia lactiflora Pall.* (Bai Shao, *Paeoniae Radix Alba*), *Glycyrrhiza uralensis Fisch. ex DC.* (Gan Cao, *Glycyrrhizae Radix et Rhizoma*), *Tetradium ruticarpum (A. Juss.) T. G. Hartley* (Wu Zhu Yu, *Evodiae Fructus*), and *Coptis chinensis Franch.* (Huang Lian, *Coptidis Rhizoma*) (**Figure 1**).

SNP comes from the classic medical book “Treatise on Febrile Diseases,” written by Zhong-Jing Zhang of the Eastern Han Dynasty. According to TCM theory, not only can it soothe the liver and regulate the stomach, but it can also have the effect of relieving the stagnation of the yang and regulate the Qi. ZJP, which is from the “Danxi Heart Law,” written by Zhen-Heng Zhu and others in the Yuan Dynasty, is based on the TCM theory of yin and yang. Coptis chinensis Franch. (Huang Lian, *Coptidis Rhizoma*) and Tetradium ruticarpum (A. Juss.) T. G. Hartley (Wu Zhu Yu, *Evodiae Fructus*) together treat the liver and stomach and play the role of acrid opening and bitter down-bearing. From a modern pharmacological perspective, it can be understood that these two herbal formulae can promote and coordinate the movement of the esophagus to the proximal small intestine by affecting the gastrointestinal myenteric plexus dopamine D2 receptor or by stimulating the 5-HT4 receptor of the gastrointestinal myenteric plexus. There are not many kinds of herbs and few medicines in these two formulae. Therefore, they are often used together as SNZJD. They can be used to treat gastrointestinal diseases, to soothe the liver, clear heat, reduce adversity, and harmonize the stomach. However, the benefits of clinical use of SNZJD in the treatment of GERD remain uncertain, and its mechanisms in treating GERD are currently vague. This article aims to provide a meta-analysis and evaluation of clinical RCTs that use SNZJD alone or SNZJD plus PPI, GPA, or MPA to treat patients with GERD. Based on the evidence of this analysis, we will use network pharmacology analysis, which can analyze the chemical basis and pharmacological action of TCM at multiple levels, to explain the effective components and targets of TCM formulae and the mechanisms of disease interaction. Through the above, we aim to provide an effective reference for the clinical use of SNZJD in the treatment of GERD.

## Data and Methods

This study was registered with the International Prospective Register of Systematic Reviews (PROSPERO) (http://www.crd.york.ac.uk/PROSPERO) as CRD42018108495 and was based on the Preferred Reporting Items for System Evaluation and Meta-Analysis (PRISMA) statements (**Table S2**).

### Data Sources and Search Strategies

We searched three English electronic databases, namely the Cochrane library (http://www.cochranelibrary.com/), PubMed (https://www.ncbi.nlm.nih.gov/pubmed/), and EMBASE (http://www.embase.com/), and three Chinese electronic databases, including the China National Knowledge Infrastructure (CNKI, http://www.cnki.net/), Wan Fang Database (http://www.wanfangdata.com.cn/index.html), and Chinese Scientific Journals Database (VIP, http://vip.hbdlib.cn), from their inception until October 2018, without language restrictions. The terminology used for searching were the following keywords: ‘ “Sini powder” OR “Sini” ‘ AND ‘ “Zuojin pill” OR “Zuojin” ‘ AND ‘ “GERD” OR “Reflux Esophagitis” OR “Gastroesophageal Reflux Disease” OR “NERD” OR “RE” OR “BE” ‘ AND ‘ “randomized” OR “RCT” ‘. The detailed search strategy can be seen in (**Table S3**).

### Study Selection and Eligibility

#### Types of Studies

All studies were RCTs for assessing the efficacy and safety of the Chinese herbal formula SNZJD only or in combination with other TSM in treating GERD. Included studies were not subject to publishing restrictions. Works on non-gastroesophageal reflux diseases or other diseases were excluded. Reports with data falsification, loss, ambiguous outcome indicators, quasi-randomized control trials, animals, and basic experiments were excluded.

#### Types of Patients

Adult patients (age ≥ 18 years) were diagnosed as GERD (including RE, NERD, or BE) according to at least one of the present or past domestic and foreign accepted diagnostic criteria, such as the Chinese Gastroenterology Diagnosis and Treatment Guidelines, Chinese Expert Consensus on GERD, Guidelines of Clinical Research of New Drugs of TCM, or World Gastroenterology Organisation Global Guidelines, or due to presenting with obvious heartburn and/or reflux symptoms or other atypical GERD symptoms or extraesophageal symptoms.

#### Types of Interventions

The treatment group was treated with SNZJD alone or with adjuvant therapy, including SNZJD plus TSM therapy but excluding SNZJD plus acupuncture, cupping, moxibustion, and other traditional therapies. Participants in the control group were being treated with placebo or TSM alone. The composition of the herbal prescription SNZJD was *Bupleurum chinense DC.* (Chai Hu, *Bupleuri Radix*), *Citrus × aurantium L.* (Zhi Shi, *Aurantii Fructus Immaturus*), *Paeonia lactiflora Pall.* (Bai Shao, *Paeoniae Radix Alba*), *Glycyrrhiza uralensis Fisch. ex DC.* (Gan Cao, *Glycyrrhizae Radix et Rhizoma*), *Tetradium ruticarpum (A. Juss.) T. G. Hartley* (Wu Zhu Yu, *Evodiae Fructus*), and *Coptis chinensis Franch.* (Huang Lian, *Coptidis Rhizoma*). There were no special restrictions on the quantity and dose of herbs and no limitation on the methods of SNZJD administration. The treatment cycle lasted for at least two weeks. If only two courses of treatment were stated in the study and the treatment duration was not reported, the original author would be consulted.

#### Types of Outcome Measures

The outcome indicators of the study should include clinical efficacy, the response rate of symptom reduction, and other effects. The former was classified as healing, markedly effective, effective, and ineffective according to the corresponding domestic and international guidelines and standards. The latter could use the Reflux Diagnostic Questionnaire (RDQ), the total score of symptoms, recurrence rate, and adverse effects, etc., for analysis. RDQ contained the assessment of heartburn, substernal chest pain, acid regurgitation, and food regurgitation symptoms.

### Data Extraction

Three reviewers (Shaowei Li, Weihan Huang, and Jinming Ou) screened and extracted the data independently using a research screening design scheme. Divergences were dealt with by discussion or consensus with the fourth reviewer (Mengfen Huang). The following data were extracted: first author, year of publication, sample size, mean age, diagnosis standard, intervention measures, duration, outcome indicators, research design, and methods. In addition, three reviewers (Guojin Wu, Xiaoqian Yang, and Qipeng Wei) extracted the composition of TCM for the decoction formula and the addition or subtraction of components with symptoms.

### Statistical Methods, Risk of Bias Assessment, TSA, and Grade Evidence

The pooled RCT meta-analysis was carried out using Cochrane Collaborations software (RevMan Version 5.3 for Windows, Copenhagen: The Nordic Cochrane Centre) to calculate mean differences (MD) and 95% confidence intervals (CI). The risk ratio (RR) was used in dichotomous data, and the weighted mean differences (WMD) were used in continuous data. At the same time, according to the heterogeneity test of the included RCTs, assessed by Cochran's Q and I^2^ test statistics, different merging methods would be adopted. The fixed effects models would be chosen for meta-analysis if a severe heterogeneity did not exist (I^2^ ≤ 50%). Otherwise, a random-effects model would be used.

The Cochrane Handbook for Systematic Reviews of Interventions (Higgins and Green, 2011) was utilized to describe the risk of bias assessment of all included RCTs. The criteria mainly included: 1) random sequence generation; 2) allocation hidden design processing; 3) blinding of participants and assessors; 4) withdrawal and loss of follow-up; 5) reporting of incomplete outcome data and selective outcome; 6) other biases.

TSA 0.9 software (Wetterslev et al., 2017) was used for trial sequential analysis of the effective rate, recurrence rate, and adverse effects. The meta-analysis resulted in an increase in random error due to multiple tests. The sequential analysis of the experiment formed a cut-off value curve by correcting the random error, i.e., the TSA threshold. It was observed whether the cumulative Z-value curve crossed the traditional threshold line, the TSA threshold line, and the vertical line of RIS information (for the avoidance of false positives in the meta-analysis and termination criteria) in order to note any significant difference between the treatment group and the control group.

GRADE profiler 3.6 software (Kaminski-Hartenthaler et al., 2013) was used to evaluate the quality of evidence and to determine the importance of therapeutic evaluation indicators. Because RCTs were the highest level of evidence, we only took five degrading conditions to evaluate the evidence, namely bias risk, uncertainty, inconsistency, indirectness, and publication bias. Finally, we could summarize the outcomes as high-quality, moderate-quality, low-quality, or very low-quality to assess the level of evidence.

### Mechanisms of Network Pharmacology of SNZJD in the Treatment of GERD

To collect the chemical compounds of all the herbs contained in SNZJD, we performed a search of the Traditional Chinese Medicine Systems Pharmacology Database (TCMSP, http://tcmspw.com/index.php). According to some research (Liu et al., 2013; Zhou et al., 2019), the average oral bioavailability (OB) value is 30%, and its variability is 10–50%; the drug-like index (DL) is stable at over 0.18. We therefore used OB ≥ 50% and DL ≥ 0.18 as filtering for thresholds.

We gathered potential targets for all chemical compounds from the above database. The Uniprot database (https://www.uniprot.org/) was used to correct the target data. The keyword “Gastroesophageal Reflux” was used to search for the genes related to GERD in two sources - CeneCards (https://www.genecards.org/) and the OMIM (https://omim.org/). We used the “VennDiagram” packages in R software to find drug-disease cross targets. To systematically understand the complex relationships among compounds, targets, and diseases, Cytoscape 3.7.0 was used to construct and analyze compound–disease target networks.

After uploading the disease–drug crossover genes to the STRING database (https://string-db.org/), setting the species to be limited to “human” to obtain protein interaction data, the related core genes were analyzed and sorted in R.

Finally, the “BiocManager” and the “org.Hs.eg.db” packages from the Bioconductor website (http://www.bioconductor.org/) were installed to change symbols into Entrez IDs, and the “DOSE,” “clusterProfiler,” and “pathview” packages were installed to conduct GO (gene ontology) annotation and KEGG (Kyoto Encyclopedia of Genes and Genomes) pathway enrichment analysis in R 3.6.2 (Huang da et al., 2009).

## Results

### Retrieval Results and Baseline

**Figure 2:**
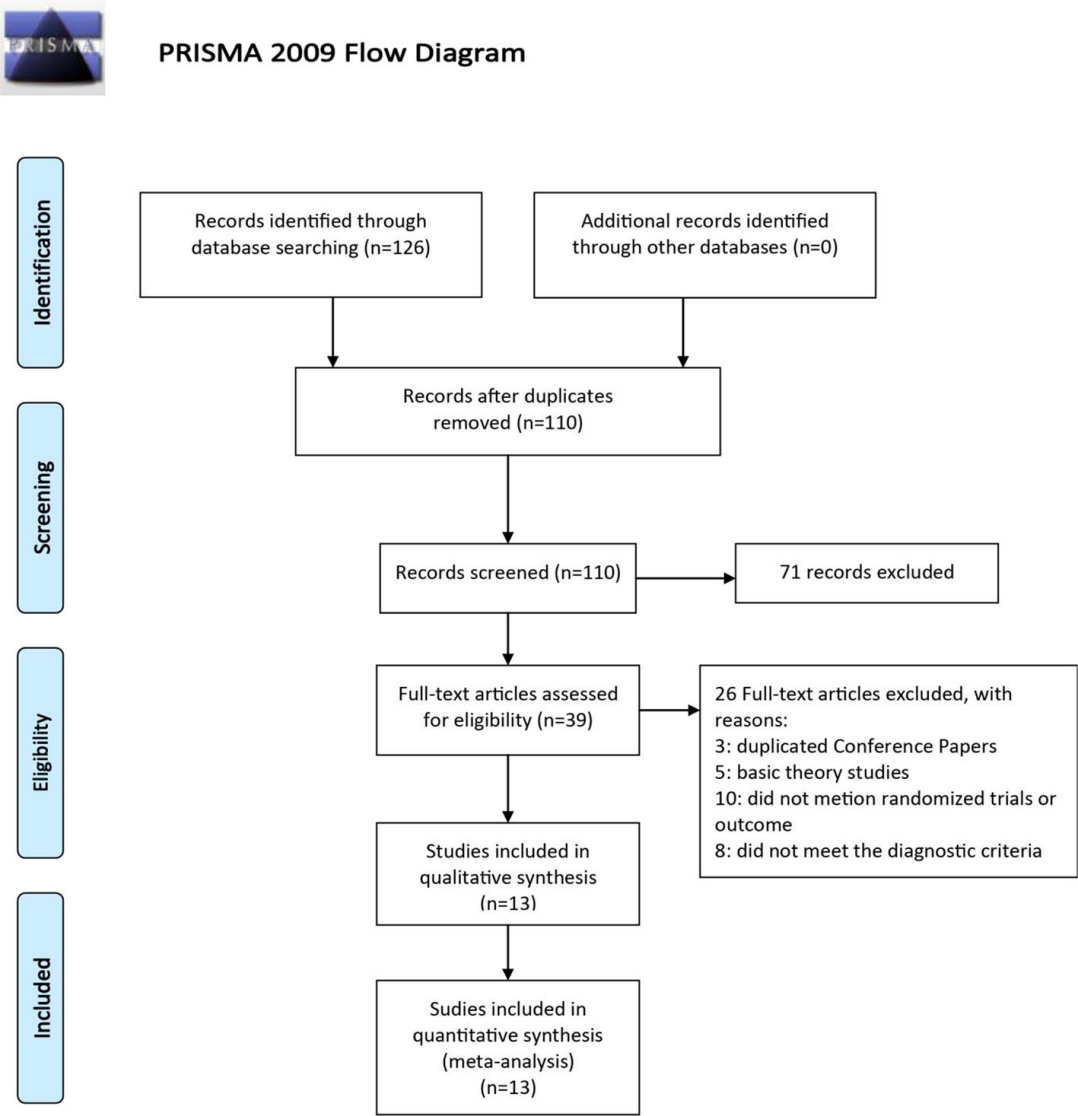
Flow diagram of eligible study selection.

A total of 126 articles were detected initially, and 16 articles were removed through eliminating duplication. A subset of 97 articles was excluded after reading the abstract carefully, including three conference papers, 67 articles where treatment was combined with other prescriptions or only one basic prescription was mentioned, three studies where treatment was combined with acupuncture, five basic theoretical studies, and 11 non-randomized trials. After reading the full text, two articles, which were animal experiments and other diagnoses, were screened out (**Figure 2**). Thirteen RCTs were selected (Xu, 2006; Gong, 2009; Tao et al., 2009; Feng, 2010; Zhu and Fang, 2011; Bi, 2013; Gan, 2014; Chu, 2015; Qin, 2015; Hou, 2016; Gao, 2017; Fan, 2018; Wang and He, 2018), all of which were in the Chinese literature. The treatment group included SNZJD and its combination therapy. The control group included PPI, GPA, MPA, or combination therapy. A total of 966 patients were included. The basic characteristics of the included studies are shown in **Table 1**. The components of SNZJD and their addition and subtraction with symptoms are shown in **Table 2**.

**Table 1:** Characteristics of included studies.

First-author (year)	Sample size （T/C）		Mean age（T/C）	Diagnosis standard	Treatment group	Control group	Duration	Outcomes	Randomized methods	Blind method	Follow-up report
(Fan, 2018)	35/35		T: 42 ± 5 C: 43 ± 5	NR	SNZJD (1 dose, 2 times/day)+A+B	A+B (Omeprazole, 20mg, 2 times/day; Domperidone Teblets, 10mg, 3 times/day)	1ms	①	RNT	NR	YES
(Hou, 2016)	50/50		T: 45.76 ± 10.06 C: 45.35 ± 10.81	NR	Modified SNZJD (200ml, 2 times/day)	A (Rabeprazole, 20mg, 2 times/day)	NR	③	NR	NR	NR
(Chu, 2015)	32/29		T: 47.28 ± 13.74 C: 50.17 ± 10.389	Expert consensus on gastroesophageal reflux disease in China in 2006	Modified SNZJD (500ml, 2 times/day)	A+B (Lansoprazole, 30mg, 2 times/day; Mosapride Citrate Dispersion, 5mg, 3 times/day)	8wks	①② ③④	MRM	NR	YES
(Feng, 2010)	32/30		T:48.5 C:48.1	Guidelines for diagnosis and treatment of reflux esophagitis in 2003	Modified SNZJD (500ml, 2 times/day)+A+B	A+B (Omeprazole, 20mg, 2 times/day; Domperidone Teblets, 10mg, 3 times/day)	4wks	①	NR	NR	NR
(Bi, 2013)	20/20		T: 44.1 ± 9.9 C: 45.3 ± 9.7	Diagnosis and treatment of reflux esophagitis (trial) in 1999	Modified SNZJD (200ml, 2 times/day)	A (Pantoprazole, 40mg, 1 times/day)	4wks	①	NR	NR	NR
(Gan, 2014)	30/30		T: 46.87 ± 12.69 C: 48.83 ± 10.64	Guidelines for diagnosis and treatment of reflux esophagitis in 2003	Modified SNZJD (300ml, 2 times/day)	A+B (Omeprazole, 20mg, 2 times/day; Domperidone Teblets, 10mg, 3 times/day)	8wks	①③④	NR	NR	NR
(Qin, 2015)	58/62		T: 42.9 ± 5.5 C: 41.5 ± 7.4	Chinese gastroenterology diagnosis and treatment guidelines in 2007	Modified SNZJD (300ml, 2 times/day)	A+B (Omeprazole, 20mg, 2 times/day; Domperidone Teblets, 10mg, 3 times/day)	3ms	①⑤	NR	NR	YES
(Zhu and Fang, 2011)	32/30		T: 32-68 C: 36-70	Expert consensus on gastroesophageal reflux disease in China in 2006	Modified SNZJD (1 dose, 2 times/day)	A (Rabeprazole, 20mg, 1 time/day)	8wks	③	NR	NR	NR
(Xu, 2006)	50/50		T:47.5 C:46.3	Diagnosis and Differential Diagnosis of Traditional Chinese Medicine	Modified SNZJD (400ml, 2 times/day)	B+C (Domperidone, 10mg, 3 times/day; Famotidine, 20mg, 2 times/day)	4wks	①	NR	NR	NR
(Wang and He, 2018)	32/31		T: 38.74 ± 9.54 C: 38.76 ± 9.57	NR	Modified SNZJD (300ml, 2 times/day)	A+B (Omeprazole, 20mg, 2 times/day; Mosapride Citrate Dispersion, 5mg, 3 times/day)	8wks	②⑤	NR	NR	NR
(Tao et al., 2009)	36	30	T: 47.12 ± 11.82 C: 46.12 ± 13.62	Chinese gastroenterology diagnosis and treatment guidelines in 2007	Modified SNZJD (200ml, 2 times/day) +A	A (Rabeprazole, 20mg, 2 times/day)	2wks	②③④	RNT	NR	YES
(Gao, 2017)	36/36		T: 41.85 ± 10.2 C: 41.65 ± 9.8	Guidelines for diagnosis and treatment of reflux esophagitis in 2003	Modified SNZJD (300ml, 2 times/day)	A+B (Rabeprazole, 10mg, 2 times/day; Mosapride Citrate Dispersion, 5mg, 3 times/day)	8wks	①②	RNT	NR	NR
(Gong, 2009)	30/30		T: 41.69 ± 14.24 C: 45.44 ± 10.43	Guidelines for diagnosis and treatment of reflux esophagitis in 2003	Modified SNZJD (400ml, 2 times/day) +A	A (Omeprazole, 20mg, 2 times/day)	8wks	①③④	RNT	NR	YES

T, treatment group; C, control group; SNZJD, Sini Zuojin Decoction; A, Proton Pump Inhibitor; B, Prokinetic Agents; C, Mucosal Protective Agents; wks, weeks; ms, months; MRM, Mechanical Random Method; RNT, Random Number Table; NR, not reported; ①, Clinical effective rate; ②, Reflux Disease Questionnaire (RDQ); ③, Total score of clinical symptoms; ④, recurrence rate; ⑤, adverse effects.

**Table 2:** SNZJD Formula components of included studies.

Study	Formula		
(Hou, 2016)	Modified SNZJD	components	Chinese Thorowax Root (Chai Hu, Bupleuri Radix) 10g; Immature Trifoliate-orange Peel (Zhi Ke, Aurantii Fructus)10g; Coptis Root, Chinese Goldthread (Huang Lian, Coptidis Rhizoma) 10g; Cultibated Purple Perilla Stalk (Zi Su Geng, Ferillae Folium) 10g; Officinal magnolia bark (Hou Po, Magnoliae Officinalis Cortex) 10g; White Peony Root (Bai Shao, Paeoniae Radix Alba) 20g; Largehead Atractylodes Rh (Bai Zhu, Atractylodis Macrocephalae Rhizoma) 15g; Mongolian Snakegourd Fruit (Gua Lou, Trichosanthis Fructus) 15g; Medcinal Evodia Fruit (Wu Zhu Yu, Euodiae Fructus) 3g
		addition and subtraction with symptom	Acid regurgitation: Cuttlebone Sepium (Hai Piao Xiao, Sepiae Endoconcha) 10g Abdominal distention: Daikon, Radish Seed (Lai Fu Zi, Raphani Semen) 15g Nausea and vomiting: Pinellia Ternata (Ban Xia, Pinelliae Rhizoma) 9g
(Fan, 2018)	SNZJD	components	Chinese Thorowax Root (Chai Hu, Bupleuri Radix) 10g; White Peony Root (Bai Shao, Paeoniae Radix Alba) 10g; Immature Fruit of the Bitter Orange (Zhi Shi, Aurantii Fructus Immaturus) 10g; Licorice Root (Gan Cao, Glycyrrhizae Radix et Rhizoma) 10g; Coptis Root, Chinese Goldthread (Huang Lian, Coptidis Rhizoma) 9g; Medcinal Evodia Fruit (Wu Zhu Yu, Euodiae Fructus) 3g
		Addition	Nutgrass Galingale Rhizome (Xiang Fu, Cyperi Rhizoma ); Inula Flower (Xuan Fu Hua, Inulae Flos); Common Gardenia Fruit (Shan Zhi Zi, Gardeniae Fructus); Hematite (Dai Zhe Shi, Haematitum)
(Chu, 2015)	Modified SNZJD	components	Chinese Thorowax Root (Chai Hu, Bupleuri Radix)6g; Coptis Root, Chinese Goldthread (Huang Lian, Coptidis Rhizoma) 3g; White Peony Root (Bai Shao, Paeoniae Radix Alba) 15g; Immature Fruit of the Bitter Orange (Zhi Shi, Aurantii Fructus Immaturus) 10g; Medcinal Evodia Fruit (Wu Zhu Yu, Euodiae Fructus) 1g; Pinellia Ternata (Ban Xia, Pinelliae Rhizoma) 10g; Cultibated Purple Perilla Stalk (Zi Su Geng, Ferillae Folium) 10g; Licorice Root (Gan Cao, Glycyrrhizae Radix et Rhizoma) 3g
		addition and subtraction with symptom	Heartburn: Baical Skullcap Root (Huang Qin, Scutellariae Radix) 10g; Weeping Forsythia Capsule (Lian Qiao, Forsythiae Fructus) 15g Nausea and vomiting: Ark Shell (Wa Leng Zi, Concha Arcae) 15g; Cuttlebone Sepium (Hai Piao Xiao, Sepiae Endoconcha) 30g Abdominal distention: Officinal magnolia bark (Hou Po, Magnoliae Officinalis Cortex) 10g; Thunberg Fritillaria Bulb (Zhe Bei Mu, Fritillariae Thunbergii Bulbus) 10g Belch: Clove Flower Bud (Ding Xiang, Caryophylli Flos) 3g; Persimmon Calyx (Shi Di, Kaki Calyx) 10g Epigastric pain: Dan-Shen Root (Dan Shen, Salviae Miltiorrhizae Radix et Rhizoma) 10g; Corydalis Yanhusuo (Yan Hu Suo, Corydalis Rhizoma) 10g; Szechwan Chinaberry Fruit (Chuan Lian Zi, Toosendan Fructus) 10g Distention and pain in chest and hypochondria: Aromatic Turmeric Root-tuber (Yu Jin, Curcumae Radix) 10g; Nutgrass Galingale Rhizome (Xiang Fu, Cyperi Rhizoma) 10g
(Feng, 2010)	Modified SNZJD	components	Chinese Thorowax Root (Chai Hu, Bupleuri Radix) 12g; Immature Trifoliate-orange Peel (Zhi Ke, Aurantii Fructus) 12g; White Peony Root (Bai Shao, Paeoniae Radix Alba) 15g; Largehead Atractylodes Rh (Bai Zhu, Atractylodis Macrocephalae Rhizoma) 15g; Coptis Root, Chinese Goldthread (Huang Lian, Coptidis Rhizoma) 12g; Medcinal Evodia Fruit (Wu Zhu Yu, Euodiae Fructus) 3g; Platycodon Grandiflorum (Jie Geng, Platycodonis Radix) 15g; Officinal magnolia bark (Hou Po, Magnoliae Officinalis Cortex) 12g; Mongolian Snakegourd Fruit (Gua Lou, Trichosanthis Fructus) 15g; Licorice Root (Gan Cao, Glycyrrhizae Radix et Rhizoma) 6g
		addition and subtraction with symptom	Acid regurgitation: Cuttlebone Sepium (Hai Piao Xiao, Sepiae Endoconcha) 12g; Ark Shell (Wa Leng Zi, Concha Arcae) 30g Belch: Inula Flower (Xuan Fu Hua, Inulae Flos) 10g; Hematite (Dai Zhe Shi, Haematitum) 30g Nausea and vomiting: Pinellia Ternata (Ban Xia, Pinelliae Rhizoma) 9g
(Bi, 2013)	Modified SNZJD	components	White Peony Root (Bai Shao, Paeoniae Radix Alba) 15g; Chinese Thorowax Root (Chai Hu, Bupleuri Radix) 9g; Immature Fruit of the Bitter Orange (Zhi Shi, Aurantii Fructus Immaturus) 9g; Coptis Root, Chinese Goldthread (Huang Lian, Coptidis Rhizoma) 10g; Licorice Root (Gan Cao, Glycyrrhizae Radix et Rhizoma) 6g; Medcinal Evodia Fruit (Wu Zhu Yu, Euodiae Fructus) 3g
		addition and subtraction with symptom	Acid regurgitation: Cuttlebone Sepium (Hai Piao Xiao, Sepiae Endoconcha) 15g Epigastric pain, Distention and pain in chest and hypochondria: Szechwan Chinaberry Fruit (Chuan Lian Zi, Toosendan Fructus) 10g Belch: Inula Flower (Xuan Fu Hua, Inulae Flos) 10g Abdominal distention: Immature Tangerine Peel (Qing Pi, Citri Reticulatae Pericarpium Viride) 10g Bitter taste, and yellow greasy tongue coating: Baical Skullcap Root(Huang Qin, Scutellariae Radix) 10g
(Gan, 2014)	Modified SNZJD	components	Chinese Thorowax Root (Chai Hu, Bupleuri Radix) 15g; Nutgrass Galingale Rhizome (Xiang Fu, Cyperi Rhizoma) 15g; Aromatic Turmeric Root-tuber (Yu Jin, Curcumae Radix) 15g; Immature Fruit of the Bitter Orange (Zhi Shi, Aurantii Fructus Immaturus) 15g; Coptis Root, Chinese Goldthread (Huang Lian, Coptidis Rhizoma) 3g; Medcinal Evodia Fruit (Wu Zhu Yu, Euodiae Fructus) 5g; Pinellia Ternata (Ban Xia, Pinelliae Rhizoma) 15g; Officinal magnolia bark (Hou Po, Magnoliae Officinalis Cortex) 15g; Ark Shell (Wa Leng Zi, Concha Arcae) 20g; Cuttlebone Sepium (Hai Piao Xiao, Sepiae Endoconcha) 20g; White Peony Root (Bai Shao, Paeoniae Radix Alba) 10g; Licorice Root (Gan Cao, Glycyrrhizae Radix et Rhizoma) 3g
		addition and subtraction with symptom	NR
(Qin, 2015)	Modified SNZJD	components	Chinese Thorowax Root (Chai Hu, Bupleuri Radix) 10g; White Peony Root (Bai Shao, Paeoniae Radix Alba) 15g; Immature Fruit of the Bitter Orange (Zhi Shi, Aurantii Fructus Immaturus) 15g; Licorice Root (Gan Cao, Glycyrrhizae Radix et Rhizoma) 6g; Coptis Root, Chinese Goldthread (Huang Lian, Coptidis Rhizoma) 10g; Medcinal Evodia Fruit (Wu Zhu Yu, Euodiae Fructus) 3-6g
		addition and subtraction with symptom	Asthenia: Codonopsis Root (Dang Shen, Codonopsis Radix) 12g Acid regurgitation, and Heartburn: Cuttlebone Sepium (Hai Piao Xiao, Sepiae Endoconcha) 30g Distention and pain in chest and hypochondria: Officinal magnolia bark (Hou Po, Magnoliae Officinalis Cortex) 10g; Nutgrass Galingale Rhizome (Xiang Fu, Cyperi Rhizoma) 10g
(Zhu and Fang, 2011)	Modified SNZJD	components	Chinese Thorowax Root (Chai Hu, Bupleuri Radix)10g; Red Peony Root (Chi Shao, Paeoniae Radix Rubra) 10g; Immature Trifoliate-orange Peel (Zhi Ke, Aurantii Fructus) 10g; Tangerine Peel (Chen Pi, Citri Reticulatae Preicarpium) 10g; Licorice Root (Gan Cao, Glycyrrhizae Radix et Rhizoma) 10g; Coptis Root, Chinese Goldthread (Huang Lian, Coptidis Rhizoma) 3-6g; Medcinal Evodia Fruit (Wu Zhu Yu, Euodiae Fructus) 3-6g; Mongolian Dandelion Herb (Pu Gong Ying, Taraxaci Herba) 15-30g; Rhubarb Root and Rhizome (Da Huang, Radix et Rhizoma Rhei) 6-10g; Tree Peony Bark (Mu Dan Pi, Moutan Cortex) 15g; Cuttlebone Sepium (Hai Piao Xiao, Sepiae Endoconcha) 15g; Inula Flower (Xuan Fu Hua, Inulae Flos) 15g; Hematite (Dai Zhe Shi, Haematitum) 15g
		addition and subtraction with symptom	Vexation and Insomnia: Tuber Fleeceflower Stem (Ye Jiao Teng, Poygoni Multiflori Caulis); Thinleaf Milkwort Root (Yuan Zhi, Polygalae Radix); Common Gardenia Fruit (Shan Zhi Zi, Gardeniae Fructus); Szechwan Chinaberry Fruit (Chuan Lian Zi, Toosendan Fructusx) Heartburn: Corydalis Yanhusuo (Yan Hu Suo, Corydalis Rhizoma); Finger Citron Fruit (Fo Shou, Citri Sarcodactylis Fructus); Aucklandia Lappa (Mu Xiang, Aucklandiae Radix); Chinese Buckeye Seed (Suo Po Zi, Aesculi Semen) Acid regurgitation: Ark Shell (Wa Leng Zi, Concha Arcae)
(Xu, 2006)	Modified SNZJD	components	White Peony Root (Bai Shao, Paeoniae Radix Alba) 15g; Chinese Thorowax Root (Chai Hu, Bupleuri Radix) 10g; Immature Fruit of the Bitter Orange (Zhi Shi, Aurantii Fructus Immaturus) 10g; Coptis Root, Chinese Goldthread (Huang Lian, Coptidis Rhizoma) 6g; Licorice Root (Gan Cao, Glycyrrhizae Radix et Rhizoma) 6g; Medcinal Evodia Fruit (Wu Zhu Yu; Euodiae Fructus) 3g
		addition and subtraction with symptom	Asthenia: Codonopsis Root (Dang Shen, Codonopsis Radix); Largehead Atractylodes Rh (Bai Zhu, Atractylodis Macrocephalae Rhizoma) Yin deficiency: Dwarf lilyturf tuber (Mai Dong, Ophiopogonis Radix); Upright Ladybell Root (Sha Shen, Adenophorae Radix) Acid regurgitation: Ark Shell (Wa Leng Zi, Concha Arcae); Cuttlebone Sepium (Hai Piao Xiao, Sepiae Endoconcha) Upset vomiting: Bamboo Shavings (Zhu Ru, Bambusae Caulis in Taenias); Reed Rhizome (Lu Gen, Phragmitis Rhizoma) Abdominal distention: Immature Tangerine Peel (Qing Pi, Citri Reticulatae Pericarpium Viride); Daikon, Radish Seed (Lai Fu Zi, Raphani Semen) Distention and pain in chest and hypochondria: Szechwan Chinaberry Fruit (Chuan Lian Zi, Toosendan Fructus); Corydalis Yanhusuo (Yan Hu Suo, Corydalis Rhizoma)
(Wang and He, 2018)	Modified SNZJD	components	White Peony Root (Bai Shao, Paeoniae Radix Alba) 15g; Indian Buead Tuckahoe (Fu Ling, Poria) 20g; Cuttlebone Sepium (Hai Piao Xiao, Sepiae Endoconcha) 15g; Chinese Thorowax Root (Chai Hu, Bupleuri Radix) 10g; Immature Fruit of the Bitter Orange (Zhi Shi, Aurantii Fructus Immaturus) 12g; Thunberg Fritillaria Bulb (Zhe Bei Mu, Fritillariae Thunbergii Bulbus) 10g; Licorice Root (Gan Cao, Glycyrrhizae Radix et Rhizoma) 6g; Inula Flower (Xuan Fu Hua, Inulae Flos) 10g; Coptis Root, Chinese Goldthread (Huang Lian, Coptidis Rhizoma) 6g; Persimmon Calyx (Shi Di, Kaki Calyx) 15g; Medcinal Evodia Fruit (Wu Zhu Yu, Euodiae Fructus) 3g; Codonopsis Root (Dang Shen, Codonopsis Radix) 10g; Largehead Atractylodes Rh (Bai Zhu, Atractylodis Macrocephalae Rhizoma) 10g
		addition and subtraction with symptom	NR
(Tao et al., 2009)	Modified SNZJD	components	Chinese Thorowax Root (Chai Hu, Bupleuri Radix) 10g; Immature Trifoliate-orange Peel (Zhi Ke, Aurantii Fructus) 10g; White Peony Root (Bai Shao, Paeoniae Radix Alba) 20g; Largehead Atractylodes Rh (Bai Zhu, Atractylodis Macrocephalae Rhizoma) 15g; Coptis Root, Chinese Goldthread (Huang Lian, Coptidis Rhizoma) 10g; Medcinal Evodia Fruit (Wu Zhu Yu, Euodiae Fructus) 3g; Cultibated Purple Perilla Stalk (Zi Su Geng, Ferillae Folium)10g; Officinal magnolia bark (Hou Po, Magnoliae Officinalis Cortex)10g; Mongolian Snakegourd Fruit (Gua Lou, Trichosanthis Fructus) 15g; Licorice Root (Gan Cao, Glycyrrhizae Radix et Rhizoma) 5g
		addition and subtraction with symptom	Abdominal distention and Belch: Daikon, Radish Seed (Lai Fu Zi, Raphani Semen) 15g Acid regurgitation: Cuttlebone Sepium (Hai Piao Xiao, Sepiae Endoconcha) 10g; Ark Shell (Wa Leng Zi, Concha Arcae) 15g Nausea and vomiting: Pinellia Ternata (Ban Xia, Pinelliae Rhizoma) 9g
(Gao, 2017)	Modified SNZJD	components	Chinese Thorowax Root (Chai Hu, Bupleuri Radix) 10g; White Peony Root (Bai Shao, Paeoniae Radix Alba) 15g; Immature Fruit of the Bitter Orange (Zhi Shi, Aurantii Fructus Immaturus) 12g; Licorice Root (Gan Cao, Glycyrrhizae Radix et Rhizoma) 6g; Coptis Root, Chinese Goldthread (Huang Lian, Coptidis Rhizoma) 6g; Thunberg Fritillaria Bulb (Zhe Bei Mu, Fritillariae Thunbergii Bulbus) 10g; Medcinal Evodia Fruit (Wu Zhu Yu, Euodiae Fructus) 3g; Cuttlebone Sepium (Hai Piao Xiao, Sepiae Endoconcha) 15g; Inula Flower (Xuan Fu Hua, Inulae Flos) 10g; Persimmon Calyx (Shi Di, Kaki Calyx) 15g; Indian Buead Tuckahoe (Fu Ling, Poria) 20g; Largehead Atractylodes Rh (Bai Zhu, Atractylodis Macrocephalae Rhizoma) 10g; Codonopsis Root (Dang Shen, Codonopsis Radix) 10g
		addition and subtraction with symptom	NR
(Gong, 2009)	Modified SNZJD	components	Chinese Thorowax Root (Chai Hu, Bupleuri Radix) 10g; White Peony Root (Bai Shao, Paeoniae Radix Alba) 15g; Immature Fruit of the Bitter Orange (Zhi Shi, Aurantii Fructus Immaturus) 10g; Licorice Root (Gan Cao, Glycyrrhizae Radix et Rhizoma) 6g; Coptis Root, Chinese Goldthread (Huang Lian, Coptidis Rhizoma) 6g; Medcinal Evodia Fruit (Wu Zhu Yu, Euodiae Fructus) 3g
		addition and subtraction with symptom	Acid regurgitation: Ark Shell (Wa Leng Zi, Concha Arcae) 10g; Cuttlebone Sepium (Hai Piao Xiao, Sepiae Endoconcha) 10g Upset vomiting: Aromatic Turmeric Root-tuber (Yu Jin, Curcumae Radix) 10g; Bamboo Shavings (Zhu Ru, Bambusae Caulis in Taenias) 10g Abdominal distention: Officinal magnolia bark (Hou Po, Magnoliae Officinalis Cortex) 10g; Daikon, Radish Seed (Lai Fu Zi, Raphani Semen) 10g Distention and pain in chest and hypochondria: Szechwan Chinaberry Fruit (Chuan Lian Zi, Toosendan Fructus) 10g; Corydalis Yanhusuo (Yan Hu Suo, Corydalis Rhizoma) 10g Bitter taste, and Yin deficiency: Mongolian Dandelion Herb (Pu Gong Ying, Taraxaci Herba) 15g; Dwarf lilyturf tuber (Mai Dong, Ophiopogonis Radix) 10g

NR, not reported.

### Inclusion of Research Quality Assessment

**Figure 3:**
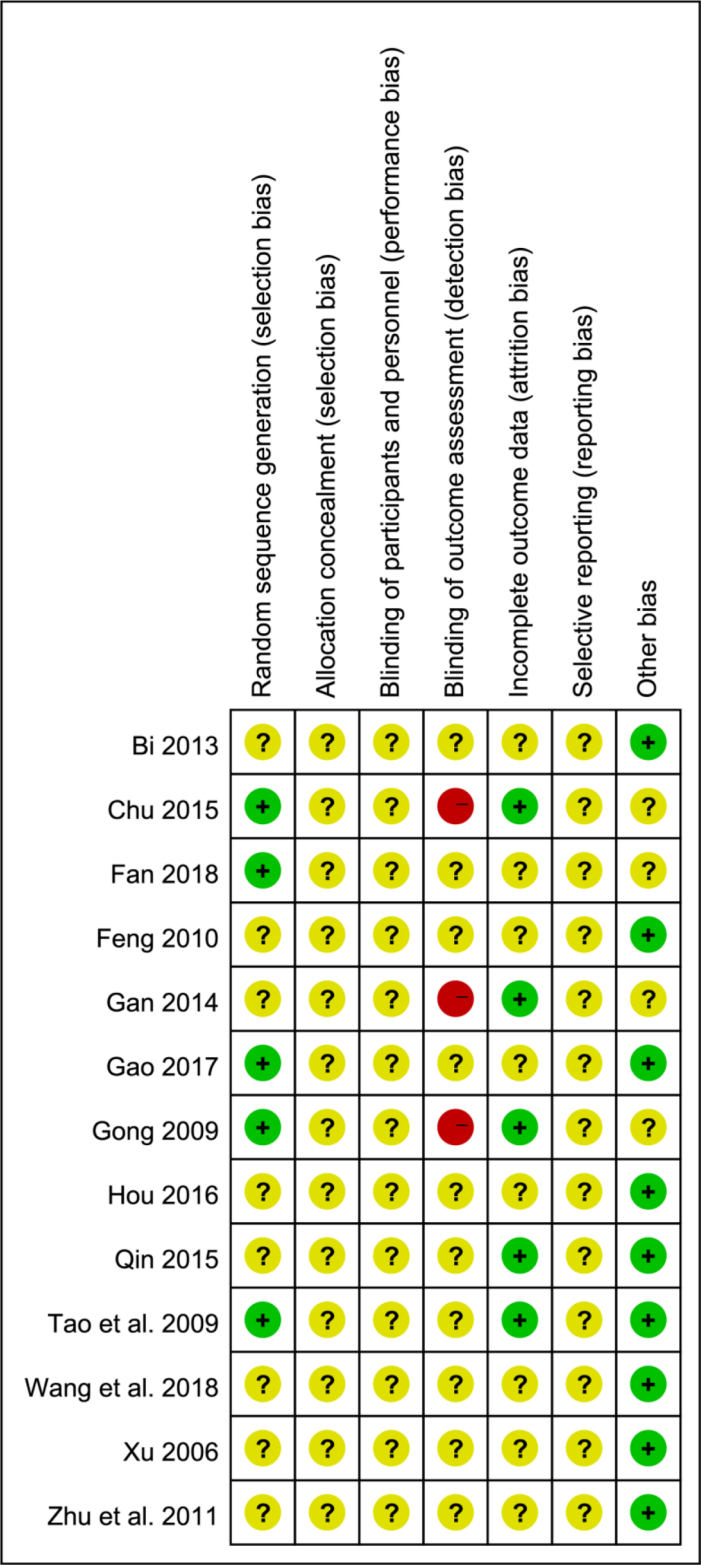
Risk of bias summary.

1) Thirteen studies included were consistent in the baseline, all studies referred to RCTs, and five studies (Gong, 2009; Tao et al., 2009; Chu, 2015; Gao, 2017; Fan, 2018) mentioned random reference by the “random number table” method or mechanical random grouping. 2) All studies clearly had no “distribution hidden” method. 3) The “blind method” of all research designs was unknown. Six studies (Gong, 2009; Tao et al., 2009; Gan, 2014; Chu, 2015; Qin, 2015) reported “follow-up.” 4) One study (Qin, 2015) reported cases of loss of follow-up. 5) There were too few indicators in some of included studies, raising doubts about selective reporting. The included studies were thus low-quality studies (**Figure 3**).

### Evaluation of Outcome Measures

#### Clinical Effective Rate

##### SNZJD versus TSM

**Figure 4:**
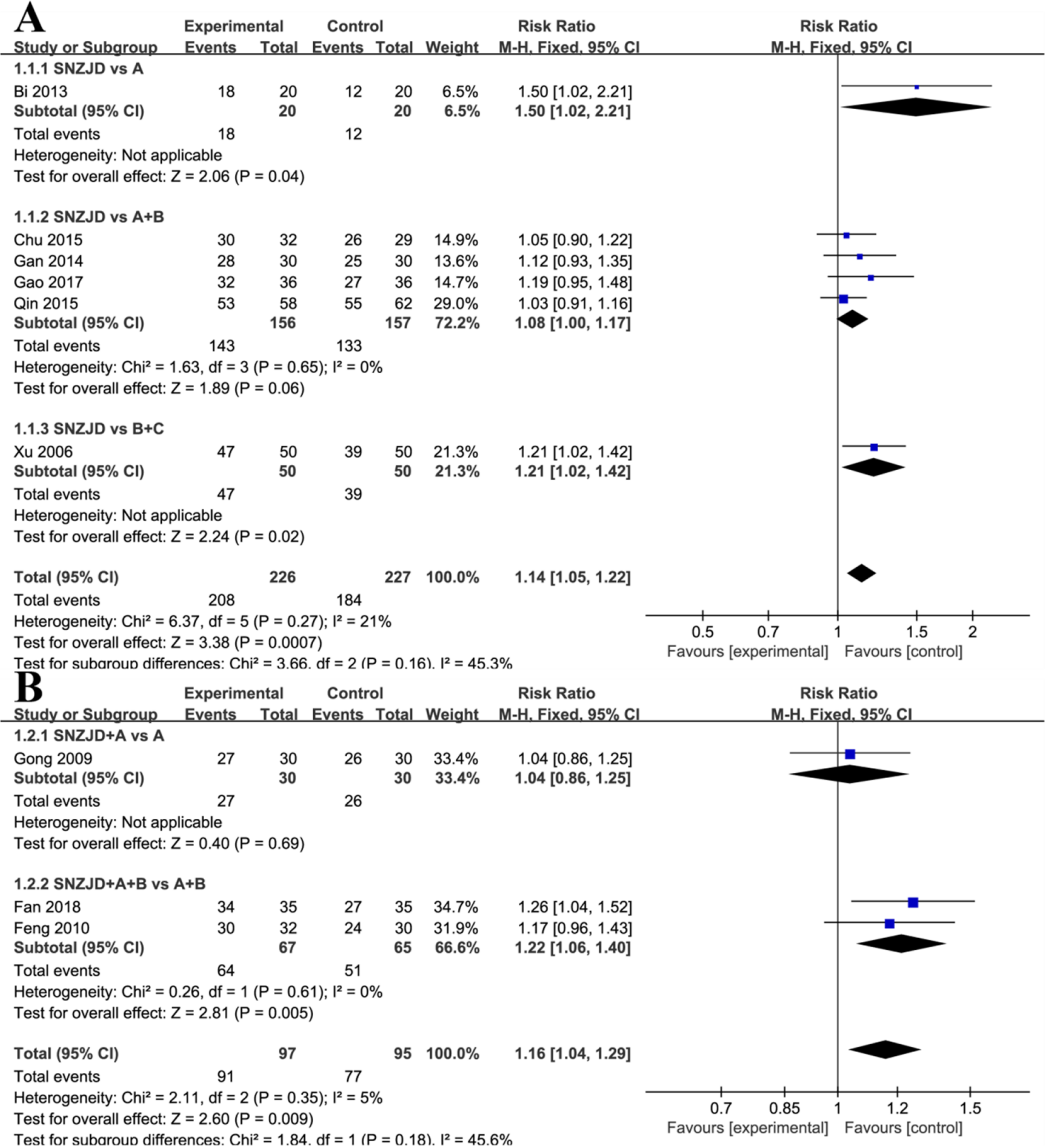
Subgroup analysis of clinical effective rate: **(A)** SNZJD vs. TSM; **(B)** SPTSM vs. TSM. SNZJD, Sini Zuojin decoction; TSM, traditional stomach medicines; SPTSM, Sini Zuojin decoction plus traditional stomach medicines.

Six studies (Xu, 2006; Bi, 2013; Gan, 2014; Chu, 2015; Qin, 2015; Gao, 2017) with a total of 453 patients provided the data for SNZJD used alone that were compared with the data of TSM. As low heterogeneity was found in this meta-analysis (Chi^2^ = 6.37, P = 0.27, I^2^ = 21%), a model of fixed effects was conducted for meta-analysis to estimate the relative risk (RR). There was a statistically significant difference in clinical effective rate between the SNZJD alone group and the TSM group, which showed favorable effects of the experimental group with SNZJD on the clinical effective rate (RR = 1.14, 95% CI [1.05, 1.22], P = 0.0007, **Figure 4A**) compared with the control group. However, since the inconsistency consisted of the experimental and control groups, owing to their different types of treatments, a subgroup analysis was also performed between different TSM groups, which comprised three subgroups: SNZJD versus PPI+GPA, SNZJD versus GPA+MPA, and SNZJD versus PPI. There was a statistically significant difference in clinical effective rate in SNZJD versus PPI but no statistically significant difference in SNZJD versus PPI+GPA (RR = 1.50, 95% CI [1.02, 2.21], P = 0.04; RR = 1.08, 95% CI [1.00, 1.17], P = 0.06, **Figure 4A**). Another result showed that treatment was more effective in the SNZJD alone group than in the GPA+MPA group and also that SNZJD+PPI+GPA had a better therapeutic effect compared with PPI+GPA (RR = 1.21, 95% CI [1.02, 1.42], P = 0.02, **Figure 4A**).

##### SPTSM versus TSM

Three studies (Gong, 2009; Feng, 2010; Fan, 2018) compared the effect of SPTSM with the effect of TSM in 192 patients with GRED. Low heterogeneity was found (Chi^2^ = 2.11, P = 0.35, I^2^ = 5%). Thus, a model of fixed effects was conducted, showing that there was a significant difference between the SPTSM group and the TSM group (RR = 1.16, 95% CI [1.04, 1.29], P = 0.009, **Figure 4B**). A subgroup analysis indicated that there was no statistically significant difference in SNZJD+PPI+GPA versus PPI+GPA (RR = 1.04, 95% CI [0.86, 1.25], P = 0.69, **Figure 4B**) but that there was a statistically significant difference in SNZJD+PPI versus PPI (RR = 1.22, 95% CI [1.06, 1.40], P = 0.005, **Figure 4B**).

#### Heartburn, Substernal Chest Pain, Acid Regurgitation, and Food Regurgitation

**Figure 5:**
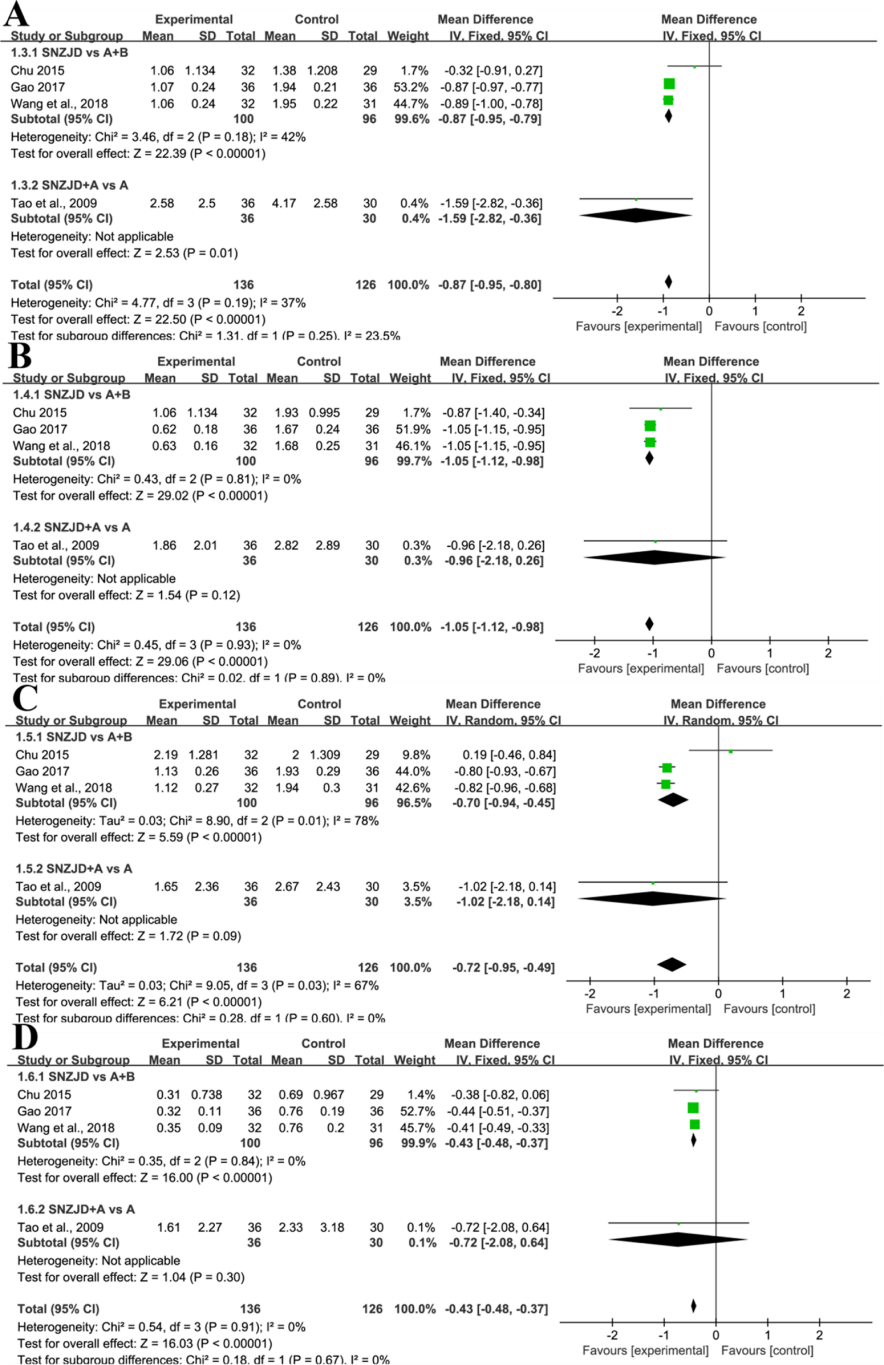
Subgroup analysis of **(A)** Heartburn, **(B)** Substernal Chest Pain, **(C)** Acid Regurgitation, **(D)** Food Regurgitation between the SNZJD vs. TSM group and SPTSM vs. TSM group (SNZJD, Sini Zuojin decoction; TSM, traditional stomach medicines; SPTSM, Sini Zuojin decoction plus traditional stomach medicines).

Four trials (Tao et al., 2009; Chu, 2015; Gao, 2017; Wang and He, 2018) evaluated values on the changes of heartburn, substernal chest pain, acid regurgitation, and food regurgitation. ① Due to a moderate homogeneity in heartburn (Chi^2^ = 4.77, P = 0.19, I^2^ = 37%), we set a fixed-effects model. As shown in **Figure 5A**, the experimental group with SNZJD was superior in easing a heartburn symptom (MD = -0.87, 95% CI [-0.95, -0.80], P < 0.0001). A subgroup analysis indicated that there was a statistically significant difference in SNZJD versus PPI+GPA and SNZJD+PPI versus PPI (MD = -0.87, 95%; CI [-0.95, -0.79], P < 0.0001; MD = -1.59, 95% CI [-2.82, -0.36], P = 0.01, **Figure 5A**). ② Analysis of substernal chest pain showed that the overall heterogeneity test was Chi^2^ = 0.45, P = 0.93, I^2^ = 0%, indicating low heterogeneity. With a fixed-effects model, the experimental group with SNZJD had an advantage in relieving pain (MD = -1.05, 95% CI [-1.12, -0.98], P < 0.0001, **Figure 5B**). A subgroup analysis that compared the effect of SNZJD used alone with the effect of TSM showed a significant difference (MD = -1.05, 95% CI [-1.12, -0.98], P < 0.0001, **Figure 5B**). ③ However, there was a high statistical heterogeneity and statistically significant difference in acid regurgitation assessment (Chi^2^ = 9.05, P = 0.03, I^2^ = 67%, MD = -0.72, 95% CI [-0.95, -0.49], P < 0.0001, **Figure 5C**). A subgroup analysis also showed an obvious difference in efficacy between the SNZJD alone group and control group (MD = -0.70, 95% CI [-0.94, -0.45], P < 0.0001, **Figure 5C**). ④ In food regurgitation of RDQ, low statistical heterogeneity was indicated between trials (Chi^2^ = 0.54, P = 0.9, I^2^ = 0%). The experimental group could effectively alleviate food regurgitation symptoms (MD = -0.43, 95% CI [-0.48, -0.37], P < 0.0001, **Figure 5D**). The meta-analysis demonstrated that there was a significant difference in SNZJD versus PPI+GPA but no significant difference in SNZJD+PPI versus PPI (MD = -0.43, 95% CI [-0.48, -0.37], P < 0.00001; MD = -0.72, 95% CI [-2.08, -0.64], P = 0.003, **Figure 5D**).

#### Symptom Total Score

##### SNZJD versus TSM

**Figure 6:**
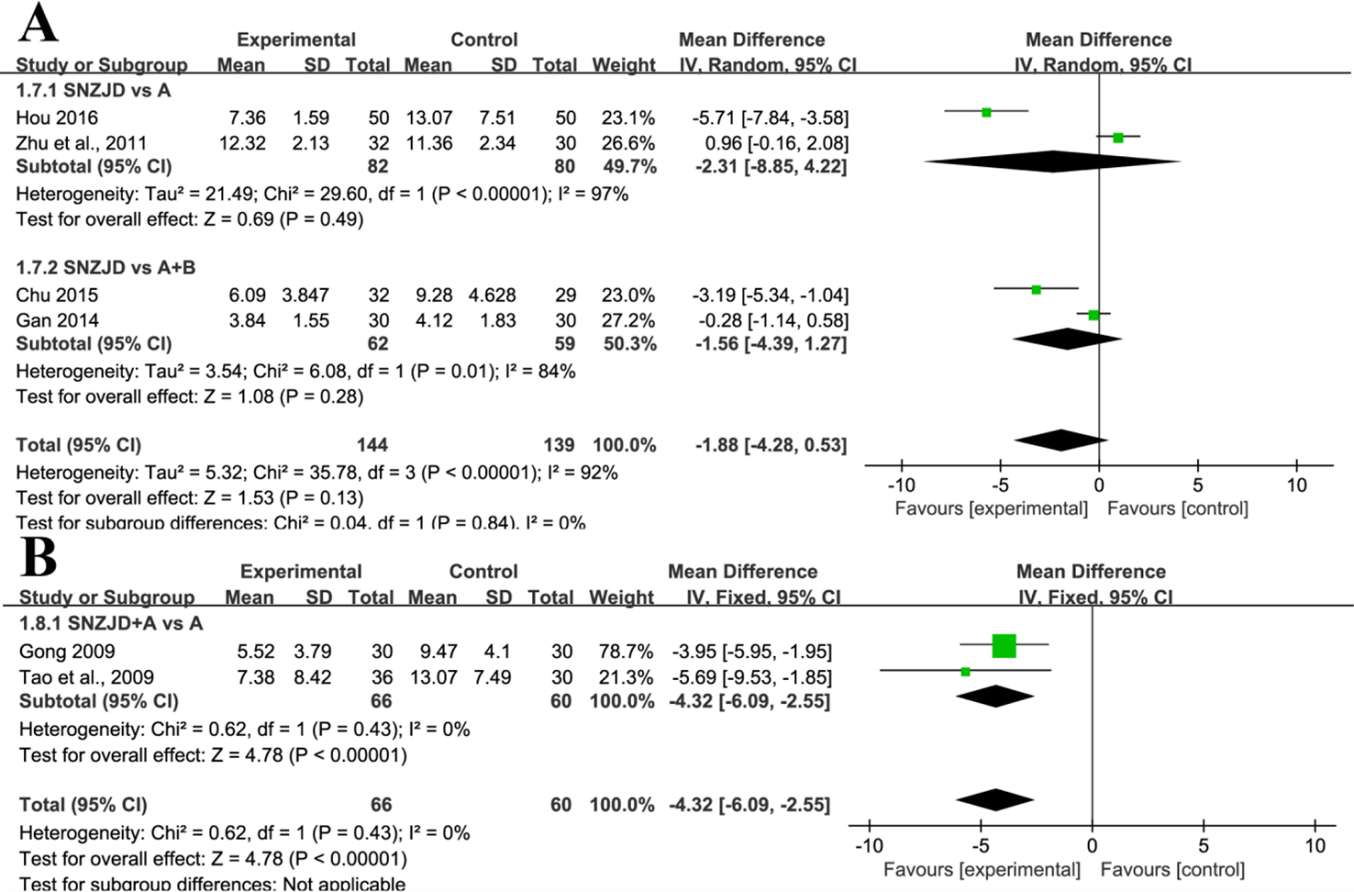
Subgroup analysis of symptom total score: **(A)** SNZJD vs. TSM; **(B)** SPTSM vs. TSM. SNZJD, Sini Zuojin decoction; TSM, traditional stomach medicines; SPTSM, Sini Zuojin decoction plus traditional stomach medicines.

Four studies (Zhu and Fang, 2011; Gan, 2014; Chu, 2015; Hou, 2016), with a total of 283 patients, compared the effect of SNZJD used alone with the effect of traditional gastric drugs by estimating the reduction of total symptom score. The studies manifested a large heterogeneity in results (Chi^2^ = 35.78, P < 0.00001, I^2^ = 92%). Performing a random-effects model, we found that there was no significant difference between the two groups (MD = -1.88, 95% CI [-4.28, 0.53], P = 0.13, **Figure 6A**). In the subgroup analysis, there was also no significant difference in SNZJD versus PPI and SNZJD versus PPI+MPA (MD = -2.31, 95% CI [-8.85, 4.22], P = 0.49; MD = -1.56, 95% CI [-4.39, 1.27], P = 0.28, **Figure 6A**).

##### SPTSM versus TSM

Two studies (Gong, 2009; Tao et al., 2009) reported the comparison of the total symptom score of GRED patients between SPTSM and TSM groups. The studies reported a slight effect on the results (Chi^2^ = 0.62, P = 0.43, I^2^ = 0%). Thus, with a fixed-effects model, there was a significant difference in SNZJD+PPI versus PPI (MD = -4.32, 95% CI [-6.09, -2.55], P < 0.00001, **Figure 6B**) The overall results showed that SPTSM helped to improve adverse symptoms overall to a certain extent.

#### Recurrence Rate and Adverse Effects

**Figure 7:**
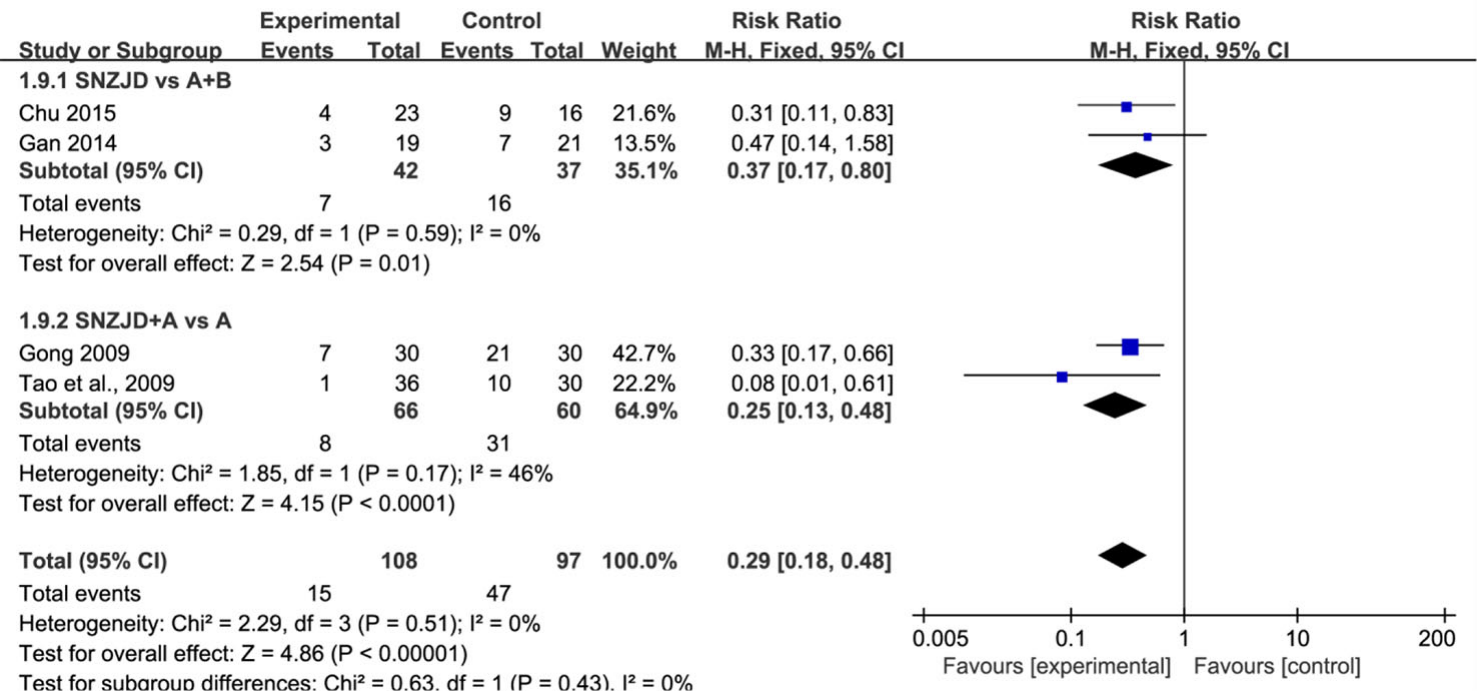
Subgroup analysis of recurrence rate between the SNZJD vs. TSM group and SPTSM vs. TSM group. SNZJD, Sini Zuojin decoction; TSM, traditional stomach medicines; SPTSM, Sini Zuojin decoction plus traditional stomach medicines.

**Figure 8:**
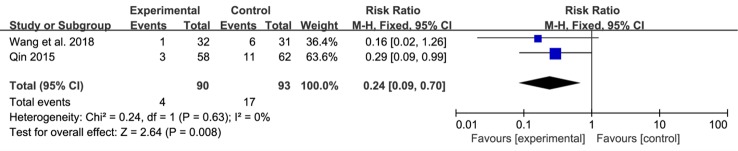
Forest plot comparison of adverse effects after treatment with SNZJD and TSM. SNZJD, Sini Zuojin decoction; TSM, traditional stomach medicines.

Recurrence rate after withdrawal was reported in four trials (Gong, 2009; Tao et al., 2009; Gan, 2014; Chu, 2015) and showed a low heterogeneity (Chi^2^ = 2.29, P = 0.51, I^2^ = 0%). With a fixed-effects model, we found that the reduction of recurrence rate in the SNZJD group was greater than that in some stomach medicine groups (RR = 0.29, 95% CI [0.18, 0.48], P < 0.00001, **Figure 7**). A subgroup analysis was conducted to evaluate results that were as homogeneous as possible. Meta-analysis showed that symptoms were significantly reduced by SNZJD alone or SNZJD plus PPI (RR = 0.37, 95% CI [0.17, 0.80], P = 0.01; RR = 0.25, 95% CI [0.13, 0.48], P < 0.0001, **Figure 7**). Adverse effects were reported in two studies (Qin, 2015; Wang and He, 2018), including dizziness, insomnia, constipation, diarrhea, and rash. The results advised that SNZJD was more likely to have fewer side effects than some other TSM (RR = 0.24, 95% CI [0.09, 0.70], P = 0.008, **Figure 8**). However, because there were only two reports in the RCTs, the safety of SNZJD remains still unknown.

#### Publication Bias and Sensitivity Analysis

If the number of studies included is less than 10, a funnel plot is of little significance, so publication bias risk assessment would not be carried out. Because of the high heterogeneity in the total symptom score, we conducted a sensitivity analysis in **Figure S1**. By excluding each study, we observed whether the outcome indicators changed significantly. The results demonstrated that the model was stable and the results were reliable.

#### TSA for the Effect of SNZJD

Based on the results of the effective rate meta-analysis, TSA calculations were performed on the effectiveness of SNZJD versus TSM and SPTSM versus TSM. The results were shown in **Figures S2A** and **S2B**; the converted Z-value penetrates the traditional threshold and TSA threshold in the first three studies. The SNZJD versus TSM group eventually passed through the expected vertical line RIS information volume, indicating that the amount of existing clinical research data could fully prove the efficacy of SNZJD in the treatment of GRED.

According to the meta-analysis of the recurrence rate, the recurrence rates of SNZJD versus TSM and SPTSM versus TSM were calculated by TSA. The results were shown in **Figure S2C** and **S2D**. The cumulative Z-value curves of both graphs crossed the traditional threshold but did not the TSA threshold. The accumulated amount of information was insufficient, indicating that the traditional meta-analysis might have a false positive result. According to the meta-analysis of the occurrence of side effects, the TSA calculation of the side effects of SNZJD versus TSM was performed: the results were shown in **Figure S2E**. The cumulative Z-value curve crossed the traditional boundary value but did not cross the TSA threshold line. The amount of information was insufficient, indicating that the traditional meta-analysis might have a false positive conclusion. Accordingly, further experiments were required to confirm efficacy.

#### GRADE Evidence for the Effect of SNZJD

Evidence in the GRADE system for the effect of SNZJD is shown in (**Table S4**). However, due to the poor methodological quality, heterogeneity, and small sample size effect, the quality of evidence was very low. Besides, because there was no evaluation of health economics, patients' willingness, and values of SNZJD, it generally, in clinical contexts, depended on doctors' medical practice, health policies, and patients' willingness to use it.

#### Network Pharmacology Results of SNZJD

##### Active Compounds Screening Results of SNZJD

According to the screening rules, a total of 74 active ingredients were retrieved from the TCMSP database, including two in Bupleuri Radix, nine in Aurantii Fructus Immaturus, seven in Paeoniae Radix Alba, 42 in Glycyrrhizae Radix et Rhizoma, nine in Euodiae Fructus, and five in Coptidis Rhizoma. The duplicate components naringenin and Mairin were removed, and a total of 72 were left.

##### Networks of Active Ingredient–Target and GERD–Target

**Figure 9:**
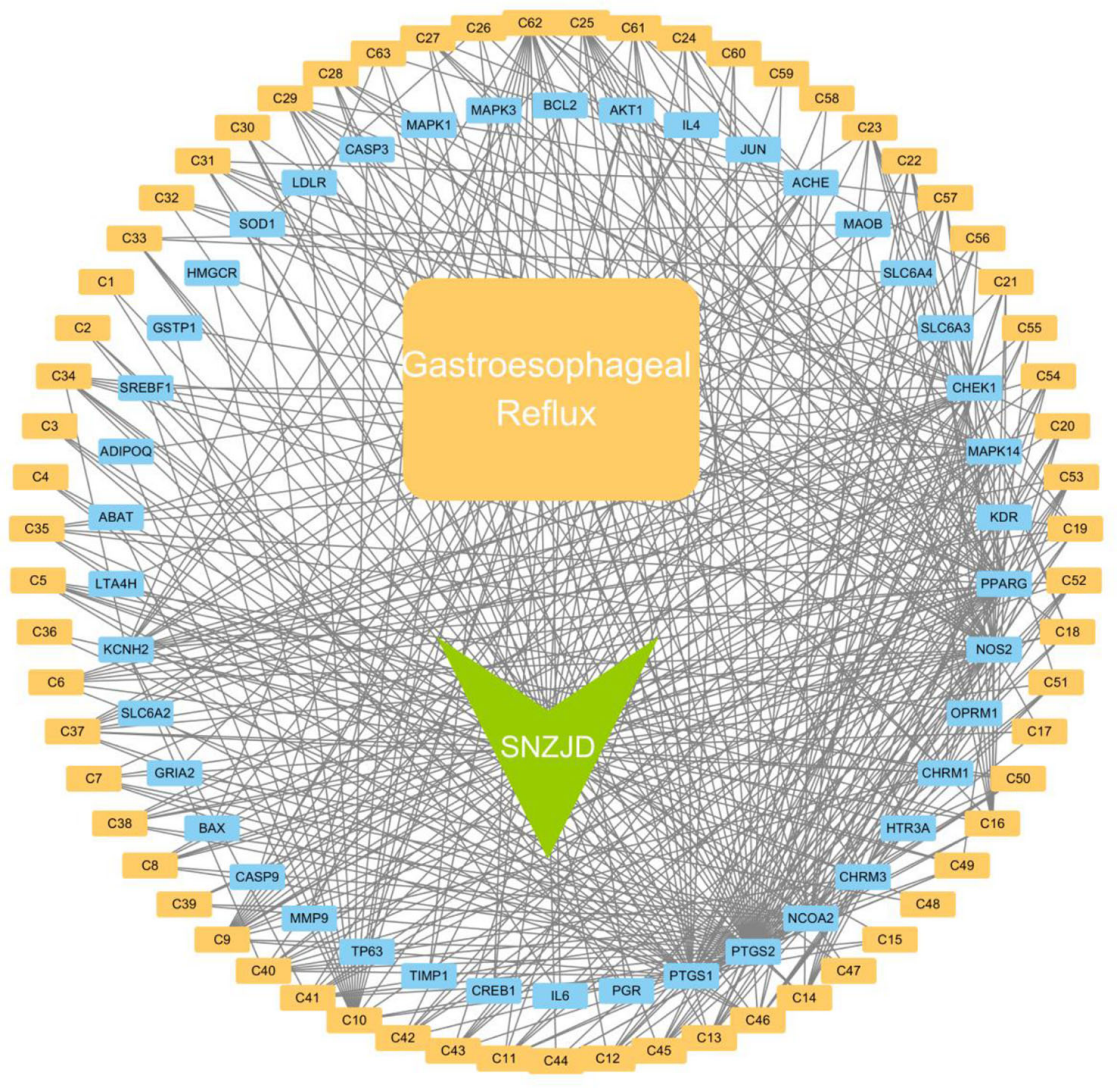
Network plot of the active compounds of SNZJD-target and GERD-target.

Among the 72 potential active components, 95 protein targets in 63 unambiguous active components (**Table 3**) were retrieved from the TCMSP database. 1878 GERD-related targets were obtained through the OMIM database and the GeneCards database. After matching the SNZJD active ingredient targets with the GERD-related targets, a total of 42 potential SNZJD targets for GERD were obtained. Cytoscape 3.7.0 software was used to construct compound–target–disease networks (**Figure 9**).

**Table 3:** The main active ingredients of SNZJD.

Active ingredients (mol)	Abbreviation	OB	DL	Active ingredients (mol)	Abbreviation	OB	DL
paeoniflorin	C1	53.87	0.79	(2R)-7-hydroxy-2-(4-hydroxyphenyl)chroman-4-one	C33	71.12	0.18
Mairin	C2	55.38	0.78	1-Methoxyphaseollidin	C34	69.98	0.64
(+)-catechin	C3	54.83	0.24	7,2',4'-trihydroxy–5-methoxy-3–arylcoumarin	C35	83.71	0.27
Cubebin	C4	57.13	0.64	8-prenylated eriodictyol	C36	53.79	0.4
Inermine	C5	75.18	0.54	Vestitol	C37	74.66	0.21
Glycyrol	C6	90.78	0.67	Gancaonin G	C38	60.44	0.39
Jaranol	C7	50.83	0.29	Gancaonin H	C39	50.1	0.78
Lupiwighteone	C8	51.64	0.37	Licoagrocarpin	C40	58.81	0.58
formononetin	C9	69.67	0.21	Glyasperins M	C41	72.67	0.59
naringenin	C10	59.29	0.21	Licoagroisoflavone	C42	57.28	0.49
glyasperin B	C11	65.22	0.44	Phaseol	C43	78.77	0.58
glyasperin F	C12	75.84	0.54	Xambioona	C44	54.85	0.87
kanzonols W	C13	50.48	0.52	dehydroglyasperins C	C45	53.82	0.37
(2S)-6-(2,4-dihydroxyphenyl)-2-(2-hydroxypropan-2-yl)-4-methoxy-2,3-dihydrofuro[3,2-g]chromen-7-one	C14	60.25	0.63	(R)-Canadine	C46	55.37	0.77
Glepidotin B	C15	64.46	0.34	Corchoroside A_qt	C47	104.95	0.78
Glypallichalcone	C16	61.6	0.19	Magnograndiolide	C48	63.71	0.19
8-(6-hydroxy-2-benzofuranyl)-2,2-dimethyl-5-chromenol	C17	58.44	0.38	palmatine	C49	64.6	0.65
Licochalcone B	C18	76.76	0.19	Evodiamine	C50	86.02	0.64
3-(2,4-dihydroxyphenyl)-8-(1,1-dimethylprop-2-enyl)-7-hydroxy-5-methoxy-coumarin	C19	59.62	0.43	hydroxyevodiamine	C51	72.11	0.71
Licoricone	C20	63.58	0.47	Evodiamide	C52	73.77	0.28
Gancaonin A	C21	51.08	0.4	Goshuyuamide I	C53	83.19	0.39
3-(3,4-dihydroxyphenyl)-5,7-dihydroxy-8-(3-methylbut-2-enyl)chromone	C22	66.37	0.41	GoshuyuamideII	C54	69.11	0.43
Glycyrin	C23	52.61	0.47	Gravacridoneshlirine	C55	63.73	0.54
licoisoflavanone	C24	52.47	0.54	N-(2-Methylaminobenzoyl)tryptamine	C56	56.96	0.26
shinpterocarpin	C25	80.3	0.73	Isosinensetin	C57	51.15	0.44
liquiritin	C26	65.69	0.74	poncimarin	C58	63.62	0.35
licopyranocoumarin	C27	80.36	0.65	isoponcimarin	C59	63.28	0.31
Glyzaglabrin	C28	61.07	0.35	neohesperidin_qt	C60	71.17	0.27
Glabridin	C29	53.25	0.47	Sinensetin	C61	50.56	0.45
Glabranin	C30	52.9	0.31	nobiletin	C62	61.67	0.52
Glabrone	C31	52.51	0.5	4-[(2S,3R)-5-[(E)-3-hydroxyprop-1-enyl]-7-methoxy-3-methylol-2,3-dihydrobenzofuran-2-yl]-2-methoxy-phenol	C63	50.76	0.39
1,3-dihydroxy-8,9-dimethoxy-6-benzofurano[3,2-c]chromenone	C32	62.9	0.53				

##### Key Targets Identified Through the STRING Database

**Figure 10:**
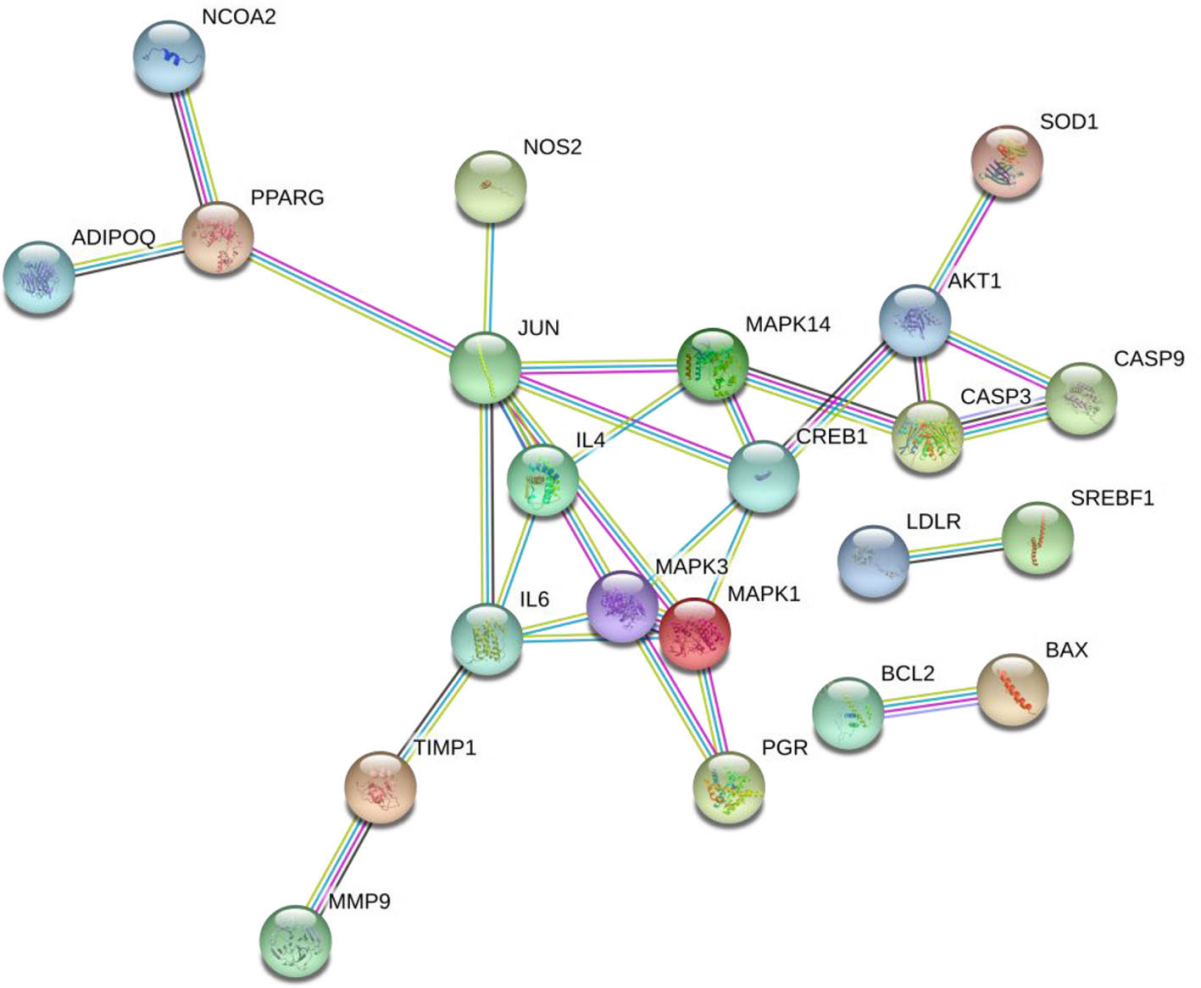
SNZJD-GERD target protein interaction network.

By uploading 42 potential targets to the STRING online database and setting the highest confidence protein participation score to more than 0.950, a protein interaction data network was obtained to get 22 related protein targets (**Figure 10**), of which the top 20 key proteins are shown in **Table S5**.

##### GO and KEGG Enrichment Analysis of the Key Targets

**Figure 11:**
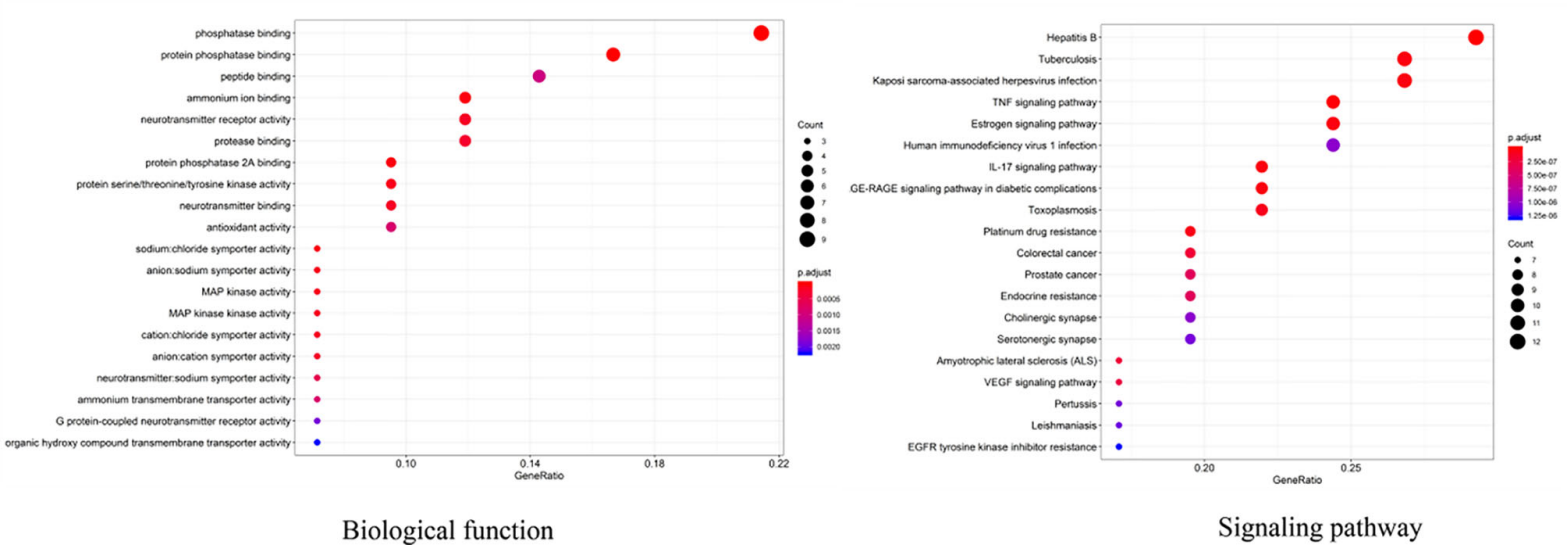
GO and KEGG enrichment analysis of the key targets of SNZJD in treatment for GERD (top 20).

To further investigate the interaction network, R 3.6.2 software was used to find that, in the GO enrichment analysis of key targets for SNZJD treatment of GERD, 82 GO entries were obtained, mainly including regulation of biosynthesis and receptor activation, such as phosphatase binding, protein phosphatase binding, neurotransmitter receptor activity, and so on. In the KEGG enrichment analysis of key targets for SNZJD treatment of GERD, a total of 129 signal pathways were identified, which were mainly found in the TNF signaling pathway, Estrogen signaling pathway, IL-17 signaling pathway, VEGF signaling pathway, and so on (**Figure 11**).

## Discussion

### Evidence Summary and Interpretation

GERD is a common digestive system disorder. However, it not only affects the digestive system; evidence suggests that it also results in other organ and tissue diseases, such as respiratory distress, laryngeal malignancy, and dental erosion (Wang et al., 2009; Parsel et al., 2019; Picos et al., 2019). Also, recent research (Dunbar et al., 2016) demonstrated that the earliest pathological changes in GERD were not chemical corrosion of the mucosal surface but inflammation in the epithelium. Therefore, the anti-inflammatory effect of some Chinese herbal medicines could be a new complementary and alternative direction for recurrent GERD that is difficult to control by PPI.

Based on the collection of many medical records, this study summarized the law of medication and evaluated the effectiveness and safety of the decoction used for GERD treatment. This was the first systematic evaluation of a type of TCM decoction in which data mining was utilized; the meta-analysis included 13 studies. The results showed that SNZJD or its combination with Western medicine in the treatment of gastroesophageal reflux was more effective than Western medicine alone. Compared with TSM alone, SNZJD had a certain curative effect, whereas SNZJD plus TSM was slightly more effective. In addition, the use of SNZJD alone or as an adjuvant therapy led to a significant reduction in the occurrence of the four basic clinical manifestations of GERD. Although compared with TSM, SNZJD used alone showed no significant difference in reducing total symptom score, SPTSM could significantly reduce total symptom score. Besides, SNZJD or its combination with TSM might prevent further reflux phenomena compared with controls after withdrawal. In the results of the statistical data, its effective rate of RR was 1.16, the MD values of reflux symptoms were - 0.87, - 1.05, - 0.72, - 0.43, and - 4.32, and the recurrence rate and safety result in RR were 0.29 and 0.24, which showed that the treatment of spleen and stomach diseases with a combination decoction of SNP and ZJP could relieve the symptoms of acid reflux, food reflux, and burning sensation more effectively. Modern research (Muluye et al., 2014; Chen and Wu, 2015) has also found that many TCMs have anti-inflammatory and anti-bacterial properties; they also improve intestinal flora. However, the TSA results showed a lack of conclusive evidence. The effects of SNZJD on clinical efficacy and safety were uncertain.

### Main Pharmacological Mechanisms

Modern pharmacological research (Shin and Seo, 2017) indicates that TCM decoctions can effectively lower the expression of inflammatory proteins in rats with reflux disease and also lower inflammatory mediators and the activation of cytokines nuclear factor-kappa (NF-κB) by protein expression related to the mitogen-activated protein kinase (MAPK) signaling pathway. In a network pharmacology study of ZJP (Yu et al., 2018), it was found to be mainly involved in anti-inflammatory effects. For the treatment of gastric symptoms, the conclusions indicate that it mainly passed through potential targets such as EGFR, IL-6, IL-1β, TNF-α, and MCP-1, and known CCKBR and IL-12β targets and a link target, NF-κB, to inhibit inflammatory reactions. In a study focusing on the liver in Chinese medicine theory (Li et al., 2013), it was shown that SNP had a good effect on digestive diseases such as colitis, constipation, and irritable bowel syndrome. Modern studies (Chang et al., 2016) have also demonstrated that the improvement of SNP can effectively improve gastrointestinal motility disorders. Biochemically, the monarch herbs of SNZJD, Rhizoma Coptidis contained berberine, and Radix Bupleuri, contain volatile oil, saikosaponin and polysaccharide, etc. For other auxiliary medicine, the main active ingredient of Fructus Immaturus Citri Aurantii is rich in Hesperidin, neohesperidin, naringin, and synephrine. Radix Glycyrrhizae contains Flavonoids and triterpenoid saponins, such as glycyrrhizin, Fml00, liquiritigenin, etc. Radix Albus Paeoniae Lactiflorae consists of Paeoniflorin as the principal ingredient, Frucus Evodiae Rutaecarpae is rich in volatile oil and evodiamine, etc.

Based on current evidence, further pharmacological analysis was performed by including SNZJD-compliant potential active ingredients and GERD-related targets in this study. Due to the OB and DL values, a total of 72 potential components were found, of which 63 were clearly related to effective treatment of GERD, such as Paeoniflorin, Evodiamine, Sainfuran, neoesperidin_qt, Liquiritin, and palmatine. Huanglian was commonly used in clinical bactericidal and anti-inflammatory drugs. Palmatine (Wang et al., 2017) was the active ingredient of Huanglian, which could increase PGE2 and decrease PAF, prevent acetic acid-induced ulcers and achieve digestive protection. Both Wuzhuyu's methanol/ethanol extract and decoction had an anti-ulcer and anti-inflammatory effect and an inhibitory effect on basic acid secretion in rats, improving acid suppression in combination with Rhizoma Coptidis (Yuan et al., 2013). Studies (Ko et al., 2007; Shen et al., 2019) also showed that evodiamine could inhibit inflammation by regulating NF-κB signaling and NLRP3 inflammasome, balance intestinal flora, and potentially prevent ulcerative colitis (UC) induced by dextran sodium sulfate (DSS). Research (Huo et al., 2011) implied that bupleurum chinense could effectively promote the release of motilin and gastrin and improve functional dyspepsia. The active ingredient in bupleurum chinense excites gastrointestinal smooth muscle, inhibits gastric acid secretion and trypsin, and exhibits anti-inflammatory, anti-acid and anti-ulcer properties. Also, in rats, licorice extract had protective and antioxidant effects on intestinal oxidative damage induced by carbon tetrachloride (CCl4) (Du et al., 2014). The licoflavone within it had the potential to regulate the inflammatory mediators and the anti-ulcer effects of amino acid metabolism (Yang et al., 2017). Furthermore, the combination of total paeoniflorin and glycyrrhiza composition Fm100 had a synergistic effect and could inhibit pain in mice; it also exhibited weak anti-inflammatory and anti-ulcer effects and had a preventive effect on stress-related ulcers in rats (Yu, 2005). Other research (Liu, 2007) indicated that Immature Bitter Orange reduced the secretion of D cells in the antrum, increased the secretion level of M cells in the duodenum, and promoted gastrointestinal peristalsis, to a certain extent. Contemporary documentation research (Zhang and Liu, 2010; Jia et al., 2017) holds that SNP regulates gastrointestinal function and exhibits anti-ulcer properties. ZJP was found to affect pain suppression, acid resistance, and anti-inflammation and had different degrees of inhibition on gastric juice, gastrin secretion, and pepsinase activity.

Therefore, combining previous research with online pharmacological results showed that the active ingredients of SNZJD often have obvious anti-inflammatory, antioxidant, acid suppression, bactericidal, and ulcer treatment effects. They also play an integral role in the digestive system. The PPI protein binding network suggested that JUN, CREB1, IL6, MAPK1, MAPK3, AKT1, and MAPK14 may be the key targets of SNZJD in the treatment of GERD, being closely related to the main anti-inflammatory effects found in modern research. Compared to the treatment of acidic corrosion, this might indicate that treating the inflammation in the epithelium that appears in early GERD should not be ignored. GO and KEGG enrichment analysis results showed that the effect of SNZJD in the treatment of GERD diseases covers multiple biological functions, such as activating conversion factors, affecting receptor activity, and promoting factor binding, involving multiple biological regulatory processes. Meanwhile, these processes might have a corresponding mechanism regulating their expression. For instance, in terms of neurotransmitter receptor activity (Travagli and Anselmi, 2016), the central nervous system and vagus nerve could regulate the activity of the upper digestive tract through a large number of neurotransmitters. Meanwhile, these processes cover multiple signaling pathways, such as the Hepatitis B, TNF signaling pathway, Estrogen signaling pathway, AGE-RAGE signaling pathway in diabetic complication, VEGF signaling pathway, and so on. For example, one study (Hsu et al., 2010) showed that chronic HBV infection is closely related to GERD. In particular, HBV carriers with high AST or TG levels are prone to induce erosive esophagitis, and this phenomenon is more common in women in terms of population probability. Moreover, further studies (Asanuma et al., 2016; Tourani et al., 2018) showed that not only can Estrogen block the development of GERD through anti-inflammatory and regulation of fat metabolism but also control the severity of chronic gastritis associated with high levels of TNF-α. In addition, there might be a complex interaction between hiv-1 protein and TNF/TNFR signaling to regulate peptic ulcer development (Pasquereau et al., 2017). Therefore, we may be able to further explore the treatment of GERD from the hepatitis pathway, the estrogen pathway, TNF pathway, and so on. It also indicated that SNZJD may treat GERD through anti-inflammation, regulating apoptosis, and regulating hormone levels. However, the general mechanisms of SNZJD in the treatment of reflux disease have not yet been thoroughly studied. In future research, we can combine chemical analysis with network pharmacology to study the pharmacological effects of complex formulations comprehensively. For one thing, the candidate target proteins and the active ingredients of the formula are predicted by analyzing the corresponding networks. For another thing, the chemical ingredients may be fully identified through experiments to confirm their presence in the formula. Therefore, further animal and clinical experiments are needed for research and exploration.

### Limitations for the Systematic Review

This study had many limitations:

1. Only Chinese RCT studies with small sample sizes were included, and there were defects in research design that resulted in the risk of heterogeneity and low quality of evidence.
2. The research had design flaws, especially because most studies focused only on results without illustrating a specific implementation of the random method, blind method, and follow-up reporting.
3. The outcome indicators were inconsistent, increasing the possibility of selective reporting.
4. There being too few reports on the adverse effects and recurrence rates might obscure the publication of negative results (as shown by the TSA results); there were indeed false-positive results in confirming the SNZJD repetition rate and safety.
5. The dosage of TCM decoction has not been standardized and unified, and the inability to determine the reasonable dosage also meant an inability to determine the optimal effectiveness and safety of use. Therefore, large-scale, high-quality RCTs are still needed to provide the best evidence.
6. The mechanism of pharmacology is not clear, especially the specific analysis of active ingredients and side effects.

Future studies need more convenient and accurate diagnostic standards so that patients can get appropriate treatment conveniently. It is also necessary to distinguish the effects of different doses on efficacy, adverse reactions, toxic and side effects, and patient acceptance to form a standard that is as unified as possible. Especially, by combining the chemical analysis and network pharmacology, the most probable active compounds may be determined, and then the chemical standardization of SNZJD can be further studied through experimental cooperation. Moreover, the analysis of the treatment cycle and relapse rate after discontinuation should be analyzed to clarify the specific mechanisms.

## Conclusion

Based on the analysis of medication regularity of 122 cases of veteran TCM practitioners, this meta-analysis, including 13 RCTs, summarized the clinical efficacy and potential mechanisms of Chinese herbal formula SNZJD in treating GERD. However, the methodological quality of the studies was too low, the risk of relapses and adverse reactions were under-reported, and related mechanisms lacked validation, so more rigorous RCTs and basic studies should be designed to assess the evidence further.

## Author Contributions

SL, SY, CL, and MH proposed research topics, designed this meta-analysis, and mainly wrote this article. WH, JO, ZH and XY screened and extracted the data. SL, QW, and GW conducted the data analysis.

## Conflict of Interest

The authors declare that the research was conducted in the absence of any commercial or financial relationships that could be construed as a potential conflict of interest.
