# Supplementary material for: Efficacy of Chinese Herbal Formula Sini Zuojin Decoction in Treating Gastroesophageal Reflux Disease: Clinical Evidence and Potential Mechanisms
Source: Front Pharmacol. 2020 Feb 27;11:76. doi: 10.3389/fphar.2020.00076 (PMC7057234; doi:10.3389/fphar.2020.00076)
Supplement: Supplementary file 1 [file Image_1.pdf]

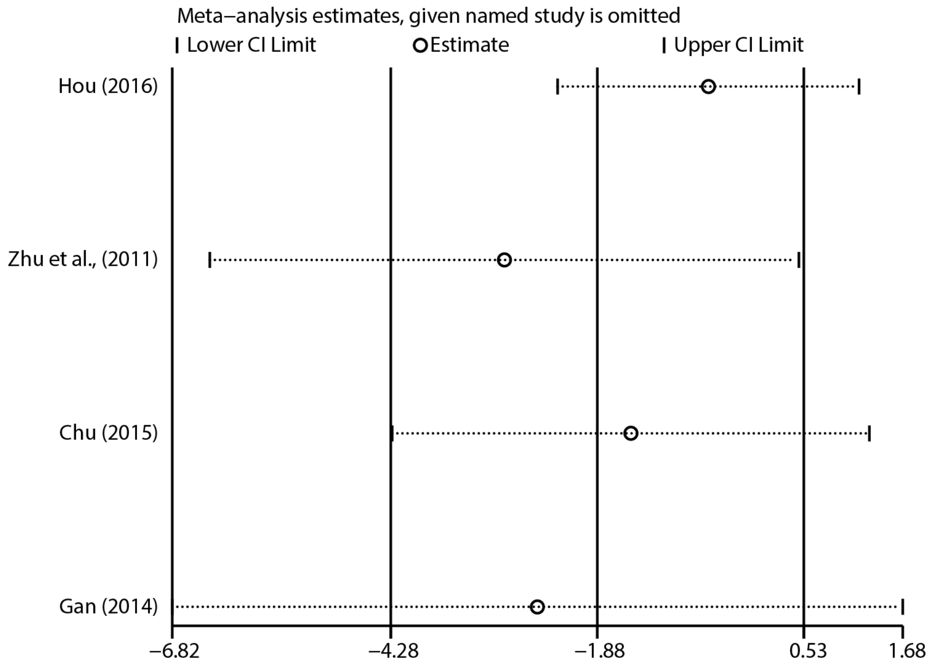

Figure S1 Sensitivity analysis of the symptom total score of SNZJD versus TSM in GERD (SNZJD, Sini Zuojin decoction; TSM, traditional stomach medicines; GERD, Gastroesophageal Reflux Disease).
