# Supplementary material for: Efficacy of Chinese Herbal Formula Sini Zuojin Decoction in Treating Gastroesophageal Reflux Disease: Clinical Evidence and Potential Mechanisms
Source: Front Pharmacol. 2020 Feb 27;11:76. doi: 10.3389/fphar.2020.00076 (PMC7057234; doi:10.3389/fphar.2020.00076)
Supplement: Supplementary file 2 [file Image_2.pdf]

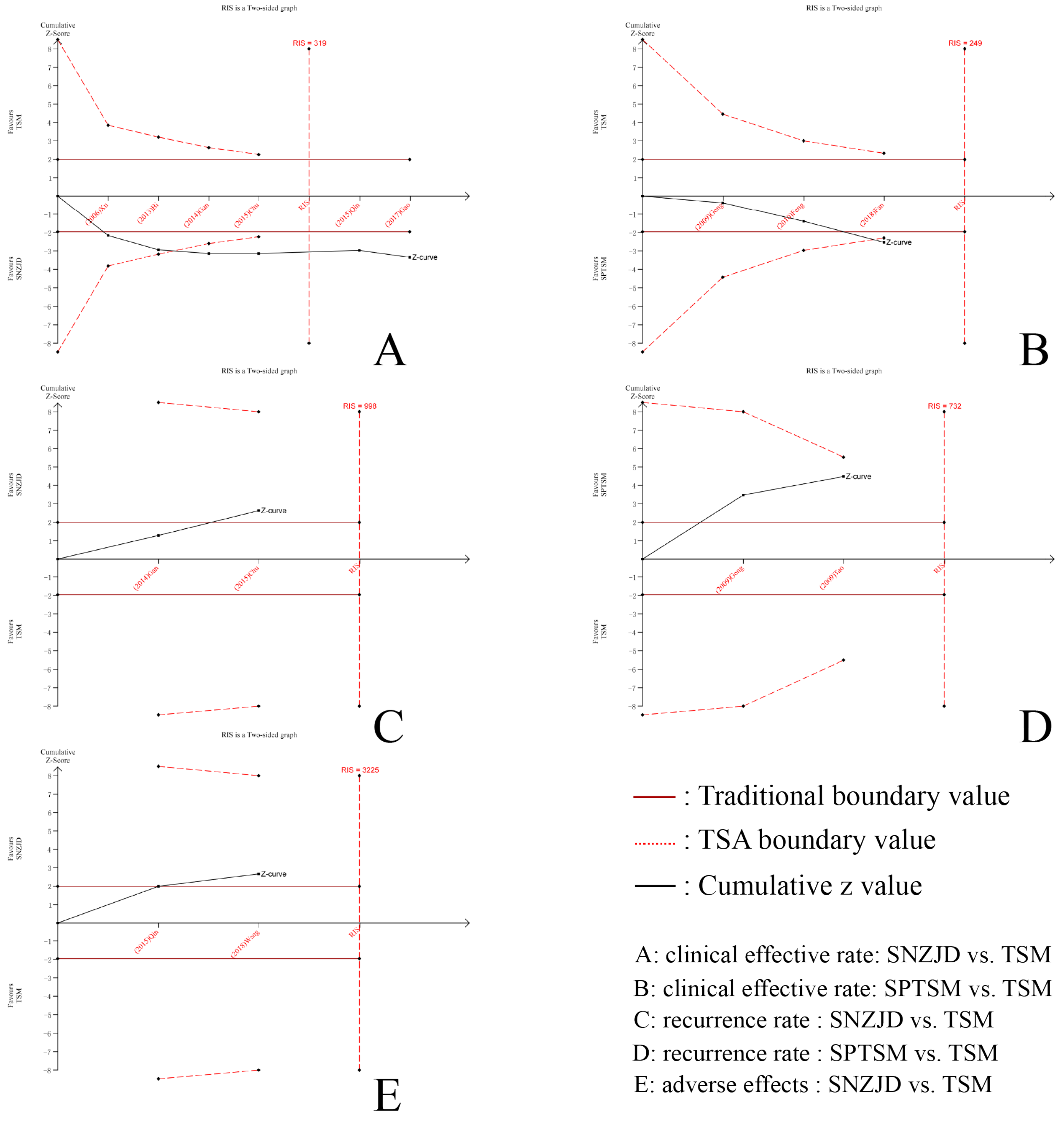

Figure S2 TSA results of main clinical indicators between SNZJD vs. TSM group and SPTSM vs. TSM group.
