# Supplementary material for: Efficacy of Chinese Herbal Formula Sini Zuojin Decoction in Treating Gastroesophageal Reflux Disease: Clinical Evidence and Potential Mechanisms
Source: Front Pharmacol. 2020 Feb 27;11:76. doi: 10.3389/fphar.2020.00076 (PMC7057234; doi:10.3389/fphar.2020.00076)
Supplement: Supplementary file 5 [file Table_3.docx]

**Search Strategies in PubMed**

#1 "Gastroesophageal Reflux"[Mesh]

#2 "Gastroesophageal Reflux" [Title/Abstract]

#3 "Acid Reflux, Gastric"[Title/Abstract]

#4 "Reflux, Gastric Acid"[Title/Abstract]

#5 "Gastric Acid Reflux Disease"[Title/Abstract]

#6 "Gastro-Esophageal Reflux"[Title/Abstract]

#7 "Gastro Esophageal Reflux"[Title/Abstract]

#8 "Reflux, Gastro-Esophageal"[Title/Abstract]

#9 "Gastroesophageal Reflux Disease"[Title/Abstract]

#10 "GERD" [Title/Abstract]

#11 "Reflux, Gastroesophageal"[Title/Abstract]

#12 "Esophageal Reflux"[Title/Abstract]

#13 "astro-oesophageal Reflux"[Title/Abstract]

#14 "Gastro oesophageal Reflux"[Title/Abstract]

#15 "Reflux, Gastro-oesophageal"[Title/Abstract]

#16 "non-erosive reflux disease"[Title/Abstract]

#17 "NERD"[Title/Abstract]

#18 "reflux esophagitis"[Title/Abstract]

#19 "RE"[Title/Abstract]

#20 "barrett esophagitis"[Title/Abstract]

#21 "BE"[Title/Abstract]

#21 #1 OR #2 OR #3 OR #4 OR #5 OR #6 OR #7 OR #8 OR #9 OR #10 OR #11 OR #12 OR #13 OR #14 OR #15 OR #16 OR #17 OR #18 OR #19 OR #20

#22 "Sini Powder"[Mesh]

#23 "Zuojin Pill"[Mesh]

#24 #4 AND #5

#25 "Sini Zuojin Decoction"[Mesh]

#26 Sini Powder[Title/Abstract]

#27 sinisan[Title/Abstract]

#28 sini[Title/Abstract]

#29 Zuojin Pill[Title/Abstract]

#30 zuojinwan[Title/Abstract]

#31 zuojin[Title/Abstract]

#32 Sini Zuojin Decoction[Title/Abstract]

#33 #22 OR #25 OR #26 OR #27 OR #28 OR #29 OR #30 OR #31 OR #32

#34 "Randomized Controlled Trials as Topic"[Mesh]

#35 "Pragmatic Clinical Trials as Topic"[Mesh]

#36 "random"[Title/Abstract]

#37 #34 OR #35 OR #36

#38 #21 AND #33 AND #37

**Search Strategies in CNKI**

#1 主题词和摘要: 胃食管反流病/全部树/全部副主题词

#2 主题词和摘要: 左金丸

#3 主题词和摘要: 四逆散

#4 主题词和摘要: 四逆左金

#5 主题词和摘要: 左金四逆

#6 主题词和摘要: 枳实

#7 主题词和摘要: 枳壳

#8 主题词和摘要: 柴胡

#9 主题词和摘要: 甘草

#10 主题词和摘要: 芍药

#11 主题词和摘要: 白芍

#12 主题词和摘要: 赤芍

#13 主题词和摘要: 黄连

#14 主题词和摘要: 吴茱萸

#15 #6 OR #7

#16 #10 OR #11 OR #12

#17 #15 AND #16 AND #8 AND #9 AND #13 AND #14

#18 #2 OR #3 OR #4 OR #5 OR #15

#19主题词或摘要: 随机

#20 #1 AND #18 AND #19
