# Supplementary material for: Efficacy of Chinese Herbal Formula Sini Zuojin Decoction in Treating Gastroesophageal Reflux Disease: Clinical Evidence and Potential Mechanisms
Source: Front Pharmacol. 2020 Feb 27;11:76. doi: 10.3389/fphar.2020.00076 (PMC7057234; doi:10.3389/fphar.2020.00076)
Supplement: Supplementary file 6 [file Table_4.doc]

Table S4. Summary of GRADE findings.

| **Quality assessment** | | | | | | | **No of patients** | | **Effect** | | **Quality** | **Importance** |
| --- | --- | --- | --- | --- | --- | --- | --- | --- | --- | --- | --- | --- |
|  |  |  |  |  |  |  |  |  |  |  |  |  |
| **No of studies** | **Design** | **Risk of bias** | **Inconsistency** | **Indirectness** | **Imprecision** | **Other considerations** | **SNZJD** | **Control** | **Relative (95% CI)** | **Absolute** |  |  |
| **Effective rate: (SNZJD vs. A)** | | | | | | | | | | | | |
| 1 | randomised trials | serious^1^ | serious^2^ | no serious indirectness | serious^3^ | reporting bias | 18/20  (90%) | 12/20  (60%) | RR 1.50 (1.02 to 2.21) | 300 more per 1000 (from 12 more to 726 more) |  VERY LOW | NOT IMPORTANT |
|  |  |  |  |  |  |  |  | 74.4% |  | 372 more per 1000 (from 15 more to 900 more) |  |  |
| **Effective rate: (SNZJD vs. A+B)** | | | | | | | | | | | | |
| 4 | randomised trials | serious^1^ | no serious inconsistency | no serious indirectness | serious^3^ | none | 143/156  (91.7%) | 133/157  (84.7%) | RR 1.08 (1 to 1.17) | 68 more per 1000 (from 0 more to 144 more) |  LOW | NOT IMPORTANT |
|  |  |  |  |  |  |  |  | 83.3% |  | 67 more per 1000 (from 0 more to 142 more) |  |  |
| **Effective rate: (SNZJD vs. B+C)** | | | | | | | | | | | | |
| 1 | randomised trials | serious^1^ | no serious inconsistency | no serious indirectness | serious^3^ | none | 47/50  (94%) | 39/50  (78%) | RR 1.21 (1.02 to 1.42) | 164 more per 1000 (from 16 more to 328 more) |  LOW | NOT IMPORTANT |
|  |  |  |  |  |  |  |  | 78% |  | 164 more per 1000 (from 16 more to 328 more) |  |  |
| **Effective rate: (SNZJD+A vs. A)** | | | | | | | | | | | | |
| 1 | randomised trials | serious^1^ | serious^2^ | no serious indirectness | serious^3^ | none | 27/30  (90%) | 26/30  (86.7%) | RR 1.04 (0.86 to 1.25) | 35 more per 1000 (from 121 fewer to 217 more) |  VERY LOW | NOT IMPORTANT |
|  |  |  |  |  |  |  |  | 86.7% |  | 35 more per 1000 (from 121 fewer to 217 more) |  |  |
| **Effective rate: (SNZJD+A+B vs. A+B )** | | | | | | | | | | | | |
| 2 | randomised trials | serious^1^ | no serious inconsistency | no serious indirectness | serious^3^ | none | 64/67  (95.5%) | 51/65  (78.5%) | RR 1.22 (1.06 to 1.4) | 173 more per 1000 (from 47 more to 314 more) |  LOW | NOT IMPORTANT |
|  |  |  |  |  |  |  |  | 78.6% |  | 173 more per 1000 (from 47 more to 314 more) |  |  |
| **Heartburn: (SNZJD vs. A+B) (Better indicated by lower values)** | | | | | | | | | | | | |
| 3 | randomised trials | serious^1^ | serious^2^ | no serious indirectness | serious^3^ | none | 100 | 96 | - | MD 0.87 lower (0.95 to 0.79 lower) |  VERY LOW | IMPORTANT |
| **Heartburn: (SNZJD+A vs. A) (Better indicated by lower values)** | | | | | | | | | | | | |
| 1 | randomised trials | serious^1^ | no serious inconsistency | no serious indirectness | serious^3^ | none | 36 | 30 | - | MD 1.59 lower (2.82 to 0.36 lower) |  LOW | IMPORTANT |
| **Substernal Chest Pain: (SNZJD vs. A+B) (Better indicated by lower values)** | | | | | | | | | | | | |
| 3 | randomised trials | serious^1^ | no serious inconsistency | no serious indirectness | serious^3^ | none | 100 | 96 | - | MD 1.05 lower (1.12 to 0.98 lower) |  LOW | IMPORTANT |
| **Substernal Chest Pain: (SNZJD+A vs. A) (Better indicated by lower values)** | | | | | | | | | | | | |
| 1 | randomised trials | serious^1^ | serious^2^ | no serious indirectness | serious^3^ | none | 36 | 30 | - | MD 0.96 lower (2.18 lower to 0.26 higher) |  VERY LOW | IMPORTANT |
| **Acid Regurgitation: (SNZJD vs. A+B) (Better indicated by lower values)** | | | | | | | | | | | | |
| 3 | randomised trials | serious^1^ | serious^2^ | no serious indirectness | serious^3^ | none | 100 | 96 | - | MD 0.7 lower (0.94 to 0.45 lower) |  VERY LOW | IMPORTANT |
| **Acid Regurgitation: (SNZJD+A vs. A) (Better indicated by lower values)** | | | | | | | | | | | | |
| 1 | randomised trials | serious^1^ | serious^2^ | no serious indirectness | serious^3^ | none | 36 | 30 | - | MD 1.02 lower (2.18 lower to 0.14 higher) |  VERY LOW | IMPORTANT |
| **Food Regurgitation: (SNZJD vs. A+B) (Better indicated by lower values)** | | | | | | | | | | | | |
| 3 | randomised trials | serious^1^ | no serious inconsistency^2^ | no serious indirectness | serious^3^ | none | 100 | 96 | - | MD 0.43 lower (0.48 to 0.37 lower) |  LOW | IMPORTANT |
| **Food Regurgitation: (SNZJD+A vs. A) (Better indicated by lower values)** | | | | | | | | | | | | |
| 1 | randomised trials | serious^1^ | serious^2^ | no serious indirectness | serious^3^ | none | 36 | 30 | - | MD 0.72 lower (2.08 lower to 0.64 higher) |  VERY LOW | IMPORTANT |
| **Symptom Total Score: (SNZJD vs. A) (Better indicated by lower values)** | | | | | | | | | | | | |
| 2 | randomised trials | serious^1^ | serious^2^ | no serious indirectness | serious^3^ | none | 82 | 80 | - | MD 2.31 lower (8.85 lower to 4.22 higher) |  VERY LOW | IMPORTANT |
| **Symptom Total Score: (SNZJD vs. A+B) (Better indicated by lower values)** | | | | | | | | | | | | |
| 2 | randomised trials | serious^1^ | serious^2^ | no serious indirectness | serious^3^ | none | 62 | 59 | - | MD 1.56 lower (4.39 lower to 1.27 higher) |  VERY LOW | IMPORTANT |
| **Symptom Total Score: (SNZJD+A vs. A) (Better indicated by lower values)** | | | | | | | | | | | | |
| 2 | randomised trials | serious^1^ | no serious inconsistency | no serious indirectness | serious^3^ | none | 66 | 60 | - | MD 4.32 lower (6.09 to 2.55 lower) |  LOW | IMPORTANT |
| **Recurrence rate: (SNZJD vs. A+B)** | | | | | | | | | | | | |
| 2 | randomised trials | serious^1^ | no serious inconsistency | no serious indirectness | serious^3^ | none | 7/42  (16.7%) | 16/37  (43.2%) | RR 0.37 (0.17 to 0.8) | 272 fewer per 1000 (from 86 fewer to 359 fewer) |  LOW | CRITICAL |
|  |  |  |  |  |  |  |  | 44.8% |  | 282 fewer per 1000 (from 90 fewer to 372 fewer) |  |  |
| **Recurrence rate: (SNZJD+A vs. A)** | | | | | | | | | | | | |
| 2 | randomised trials | serious^1^ | serious^2^ | no serious indirectness | serious^3^ | none | 8/66  (12.1%) | 31/60  (51.7%) | RR 0.25 (0.13 to 0.48) | 388 fewer per 1000 (from 269 fewer to 450 fewer) |  VERY LOW | CRITICAL |
|  |  |  |  |  |  |  |  | 51.7% |  | 388 fewer per 1000 (from 269 fewer to 450 fewer) |  |  |
| **Adverse Effects** | | | | | | | | | | | | |
| 2 | randomised trials | serious^1^ | no serious inconsistency | no serious indirectness | serious^3^ | none | 4/90  (4.4%) | 17/93  (18.3%) | RR 0.24 (0.09 to 0.7) | 139 fewer per 1000 (from 55 fewer to 166 fewer) |  LOW | IMPORTANT |
|  |  |  |  |  |  |  |  | 18.6% |  | 141 fewer per 1000 (from 56 fewer to 169 fewer) |  |  |

^1^ Poor methodological quality (method of randomized allocation, blind method, allocation concealment were not clear yet)
^2^ Substantial heterogeneity or there was no statistical significance in the results
^3^ Small sample size
