# Supplementary material for: Efficacy of Chinese Herbal Formula Sini Zuojin Decoction in Treating Gastroesophageal Reflux Disease: Clinical Evidence and Potential Mechanisms
Source: Front Pharmacol. 2020 Feb 27;11:76. doi: 10.3389/fphar.2020.00076 (PMC7057234; doi:10.3389/fphar.2020.00076)
Supplement: Supplementary file 7 [file Table_5.doc]

Table S5 Key targets of SNZJD protein interaction network for GERD treatment (top 20).

| Number | Gene Symbol | Count value |
| --- | --- | --- |
| 1 | JUN | 8 |
| 2 | CREB1 | 5 |
| 3 | IL6 | 5 |
| 4 | MAPK1 | 5 |
| 5 | MAPK3 | 5 |
| 6 | AKT1 | 4 |
| 7 | MAPK14 | 4 |
| 8 | CASP3 | 3 |
| 9 | IL4 | 3 |
| 10 | PPARG | 3 |
| 11 | CASP9 | 2 |
| 12 | PGR | 2 |
| 13 | TIMP1 | 2 |
| 14 | ADIPOQ | 1 |
| 15 | BAX | 1 |
| 16 | BCL2 | 1 |
| 17 | LDLR | 1 |
| 18 | MMP9 | 1 |
| 19 | NCOA2 | 1 |
| 20 | NOS2 | 1 |
